# Improving the estimation of educational attainment: New methods for assessing average years of schooling from binned data

**DOI:** 10.1371/journal.pone.0208019

**Published:** 2018-11-29

**Authors:** Joseph Friedman, Nicholas Graetz, Emmanuela Gakidou

**Affiliations:** 1 David Geffen School of Medicine at University of California, Los Angeles, CA, United States of America; 2 Institute for Health Metrics and Evaluation, University of Washington, Seattle, WA, United States of America; 3 Department of Demography, University of Pennsylvania, Philadelphia, PA, United States of America; Vrije Universiteit Amsterdam, NETHERLANDS

## Abstract

**Background:**

The accurate measurement of educational attainment is of great importance for population research. Past studies measuring average years of schooling rely on strong assumptions to incorporate binned data. These assumptions, which we refer to as the standard duration method, have not been previously evaluated for bias or accuracy.

**Methods:**

We assembled a database of 1,680 survey and census datasets, representing both binned and single-year education data. We developed two models that split bins of education into single year values. We evaluate our models, and compare them to the standard duration method, using out-of-sample predictive validity.

**Results:**

Our results indicate that typical methods used to split bins of educational attainment introduce substantial error and bias into estimates of average years of schooling, as compared to new approaches. Globally, the standard duration method underestimates average years of schooling, with a median error of -0.47 years. This effect is especially pronounced in datasets with a smaller number of bins or higher true average attainment, leading to irregular error patterns between geographies and time periods. Both models we developed resulted in unbiased predictions of average years of schooling, with smaller average error than previous methods. We find that one approach using a metric of distance in space and time to identify training data, had the best performance, with a root mean squared error of mean attainment of 0.26 years, compared to 0.92 years for the standard duration algorithm.

**Conclusions:**

Education is a key social indicator and its accurate estimation should be a population research priority. The use of a space-time distance bin-splitting model drastically improved the estimation of average years of schooling from binned education data. We provide a detailed description of how to use the method and recommend that future studies estimating educational attainment across time or geographies use a similar approach.

## Introduction

As a key marker of global progress, the accurate measurement of education is of great importance for population research. Education has been prioritized as a global development indicator, especially in the Millennium Development Goal 2 and Sustainable Development Goal 5 targets [1,2]. Educational attainment has also been linked to numerous health outcomes at both the individual and national level. The association between maternal education and child mortality and morbidity is especially robust, and has been the focus of much study [3–12]. Increases in education among women has also been strongly linked to reduced fertility, and subsequent decreases in maternal mortality [13–16]. Due to its predictive power across many domains, education is routinely used as a covariate in many studies estimating demographic or disease trends in the absence of complete data [17–21] or seeking to control for confounding by socio-economic status [22–24]. The accurate measurement of educational attainment is therefore of paramount importance for facilitating reliable and unbiased population research, and monitoring development targets.

Information about educational attainment is available from a large number of survey and census data sources [25–27], either in single-years of attainment or in binned levels, such as “achieved primary education,” or “some secondary education completed.” Calculating average years of schooling is a straightforward arithmetic mean when data are available in single years of attainment. However, when data are only available in binned form, assumptions about the distribution of individuals within each bin must be used. In their 1986 work, Psacharopoulos and Arriagada were among the first to propose a simple approach, which we refer to here as the “standard duration method”[28]. They calculate the average years of schooling $\bar{S}$ using *L*_*i*_, the proportion of the population who have completed the *i*^*th*^ level of school and *S*_*i*_, the standard duration of school completed by people finishing the *i*^*th*^ level, as seen below in Eq 1.

$$\bar{S}=\sum_{i}L_{i}S_{i}$$ (1)

The standard duration method therefore simply assumes a single time invariant number of completed years for all individuals in each bin, which is either equal to the typical duration of the level of schooling in question, or equal to the midpoint of the bin when the educational level is reported as incomplete. Therefore, for an educational system where primary education represents years one to five of educational attainment, an individual reporting completed primary education receives five years, and an individual reporting incomplete primary receives three. This same general formula has been used by a large number of studies including those of Barro and Lee, the UNESCO Institute for Statistics, and others [12,28–36].

The assumptions involved in the standard duration method are strong for a number of reasons. Information about incomplete attainment is often provided inconsistently, and so individuals who have completed some portion of a bin are coded down to completion of the level of education below. All individuals with tertiary education are often represented with a single code for “university,” obscuring individuals who completed tertiary education of a non-standard length, such as a Master’s or Doctoral degree. These assumptions represent a possible source of differential bias if the inaccuracies they induce are not consistent between multiple geographies and time periods. Differences in true drop-out patterns or education binning schemas between data sources could lead to attainment estimates which are not directly comparable. Furthermore, the number of bins in each data source can vary, and it is reasonable to assume increased bias would be observed in surveys with fewer bins.

Although there is an existing body of literature describing sources of bias in estimates of educational attainment, previous work has not addressed the standard duration method explicitly. Instead it has focused on identifying data that have been recorded incorrectly [37,38] or adjusting average years of schooling for the inherent quality of the education [31,39]. In this paper we seek to provide the first characterization of the bias and accuracy involved in using the standard duration method to estimate educational attainment. We also propose two models to split binned education data into single-year values and evaluate all three methods using out-of-sample predictive validity. We amassed a large database describing the distribution of years of educational attainment for a large number of countries and years. This training database, and the proposed methods, allow practitioners who need to work with binned educational attainment data to split their binned data to single years of education to minimize the bias in their estimates. The practitioner can find a list of countries and years in our training database in the S1 File, and all code is made available in the the S2 File.

## Methods

### Data sources

We compiled a database of n = 1,680 surveys and census datasets that provide education in both single-year (n = 1,518) and binned (n = 162) formats (see Table 1 for a summary by data provider and S1 File for a complete source list). The single-year datasets were acquired from a number of survey and census data providers, while the binned data were all obtained from Integrated Public Use Microdata Sample (IPUMS) datasets [27]. All single-year datasets were used to calculate the proportion of each sex-specific, five-year age group with each single-year of educational attainment from 0 to 18. We used 18 years of schooling as the top code in our analysis as it is a common choice among providers of single-year education data [40], and it is reasonable to assume that the importance of education for health diminishes greatly after the completion of 18 years, which represents 2 to 3 years of graduate education in most educational systems. All binned datasets were used to calculate the proportion of each sex-specific, five-year age group within each bin of educational attainment provided in the survey. Table 2 shows the total count of binning schemas by number of bins present in the data.

**Table 1:** Number of sources by data provider.

Data Source	Country-Years
Integrated Social Survey Programme	592
EUROBAROMETER	551
Demographic and Health Survey	217
Integrated Public Use Microdata Samples	99
World Health Survey	59
**Total**	1518

All single-year data sources used in the analysis, tabulated by count of unique country-years sources by data provider. Specific details on each dataset used in the analysis can be seen in the S1 File.

**Table 2:** Number of sources by bin number.

Number of Bins	5	6	7	8	9	10	12	13	14	15	17	18	Total
**Sources**	26	25	37	8	2	1	3	4	29	6	3	18	162

Number of binning data sources used in the analysis. Shown as counts of unique country-years by the number of bins present in the binning schema of the survey.

### Models to split bins into single year values

We developed two models to split binned education values to single-years of attainment. Instead of mapping each bin to a single average number of years of attainment, as is done in the standard duration method, we probabilistically split the bin into the proportion of individuals with each single year contained within the bin. Therefore, a bin containing individuals with at least one, but not more than four years of education, would be split into the proportion with one, two, three, and four years of attainment. This approach both allows for more nuanced estimation of average years of schooling, the study’s primary objective, and allows for the preservation of more precise information about the distribution of education within each population being measured.

The first model uses nested hierarchical mixed effects, and the second uses a metric of space-time distance to determine an optimal training dataset before using a simple averaging process. Both models are run separately for each survey and sex specific five-year age group being split. In both cases, the first step is to take the binning schema present in the survey data being split and apply it to each single-year dataset in the training sample inducing the same bins. Next, one of the models delineated below is used to predict what proportion of each bin should be allocated to each single-year of attainment within that bin. Finally, the predicted proportions are normalized to ensure internal consistency for each bin. This step is necessary to adjust for the independent estimation of the proportion within each single year, guaranteeing that the final estimates will add up to 100% of the bin of educational attainment being allocated. All code used to run the models is made available in the S2 File.

#### Nested hierarchical mixed effects model

The first model uses the full set of all available training data, after the binning schema being split has been applied to each dataset, and a set of nested hierarchical mixed effects to capture the temporal and spatial trends in the distribution of proportions within each bin. The model is shown in Eq 2.

$${logit(p}_{b,y})∼\beta_{0}+\propto_{r}+\propto_{l}+\propto_{s}+\propto_{a}+\beta_{1}(Year)$$ (2)

*p*_*b*,*y*_ represents the logit transformed proportion of individuals in bin *b* who are assigned to the single-year of education *y*. ∝_*l*_ represent random intercepts for each country, which are nested within ∝_*r*_ random intercepts for each region used in the Global Burden of Disease 2013 Study[21]. ∝_*a*_ are random five-year age-group-specific intercepts, nested within ∝_*s*_ sex-specific random intercepts. *β*_0_ is a global intercept, and *β*_1_ captures any overall secular trend.

#### Space-time distance model

The second model tests the hypothesis that geography and time are the most important determinants of the proportions within each bin, and that given the large amount of data available for use in this analysis, a more accurate prediction may be achieved by strategically subsetting the training dataset to only the most proximate data. The model first determines the distance between the survey being split, and all available datasets in the training set, using a metric of space-time distance on a 0 to 1 scale. Distance in time is defined as the difference in years between the two datasets, divided by the total range of years present in the training dataset. Distance in space is created using a set of region and super-region groupings established by the Global Burden of Disease 2013 study [21]. Data from the same country have a spatial distance of 0, data in the same region have a distance of .33, data in the same super-region have a distance of .66, and all other data have a distance of 1. The space and time distances are combined using the below formula, shown in Eq 3.

$$D_{F}={(D}_{S}*\pi )+{(D}_{T}*(1-\pi ))$$ (3)

*D*_*F*_ represents the final distance between two datasets, *D*_*S*_ represents the distance in space, *D*_*T*_ is the distance in time, and *π* is the space-time weight. If *π* has a value of 1 then the full value of *D*_*F*_ comes from spatial distance. If *π* is 0, distance in time has the full weight. For all intermediate values between 0 and 1, space and time distances are combined with relative importance. The model then keeps the *η* closest datasets and calculates the mean proportion within each bin-year using the simple formula shown below in Eq 4.

$${logit(p}_{b,y)}∼\beta_{0}$$ (4)

*β*_0_ is a global intercept used to calculate the average *p*_*b*,*y*_ or proportion of individuals in bin *b* with *y* years of educational attainment. The hyper-parameters in this model, *π* and *η*, are optimized using a grid search and out-of-sample predictive validity, as detailed below. As a sensitivity analysis we also tried testing the effect of using a space-time distance crosswalk approach that weights training data by distance, as opposed to weighting all training data points equally (S3 Table).

### Comparison to standard duration algorithm

To compare the standard duration algorithm to the two bin-splitting models defined above, we used the typical normative assumptions used by Barro and Lee [29–32], De la Fuente and Dom [34–36], and others. These include assuming that a) any individual who reports completing a level of education dropped out immediately after that level’s completion and b) individuals who report starting but not finishing a level of education drop out on average at the exact midpoint of the range of possible values.

After the space-time distance model was optimized, we used the model with the best-performing parameter set, as well as the nested hierarchical mixed effect model and the standard duration method to split the actual binned data to single year values and produce average years of schooling estimates. Differences in the estimates were analyzed to understand the implications of model selection on global trends.

### Out-of-sample predictive validity

In order to assess the performance of our two bin-splitting models, and compare them to the standard duration method, we use out-of-sample predictive validity testing [41–46]. We take the approach of artificially binning a large quantity of single year surveys in our dataset, and comparing the performance of each model in splitting bins into its component single years to the true single year proportions. We use ten-fold cross validation, which entails apportioning our database of single-year education data sources into ten equally sized sections. We iteratively ‘knock-out’ each 10% section of the data by randomly selecting and applying a binning schema from our set of binned education datasets to the single-year data. We then use the remaining 90% to predict the newly binned testing data, for which we know the true single-year proportions and evaluate the model performance. This process was repeated three times to ensure the results are not a function of idiosyncrasies in one particular test-train split, and the results were averaged [47]. Knock-outs were randomly chosen in a country and data source specific fashion, to attempt to mimics the true pattern of observed data availability, where binning patterns are almost always specific to a country and data source[46]. For the space-time distance based model, three iterations of validation were completed for each parameter set in a grid search of pairs of *π* and *η* values. We iterated over all combinations of *π* values from 0 to 1 in .1 increments, as well as *η* values of 1,2,4,6,8,12,16,20,40,60 and 80.

The outcome measures used to evaluate out-of-sample predictive validity included root mean squared error (RMSE), which represents the average model error, and median error, which highlights any prediction bias. Each statistic was calculated with respect to the models’ prediction of both average years of schooling, as well as the standard deviation of educational attainment. As the primary metric of educational attainment, average years of schooling is of special importance in establishing model performance [28]. The standard deviation of education has been used in a number of studies to assess inequality of education, and was used to evaluate the various models’ performance in estimating the full distribution of education [48–50]. As the difficulty of predicting within-bin proportions should decrease with a greater number of bins present in a schema, all predictive validity measures for each model were assessed both globally and with respect to the number of bins in the schema being split. Trends in bias and accuracy were also analyzed with respect to the magnitude of the true population mean being estimated to capture ways in which the effect of binning may vary between differently educated populations.

## Results

### Comparative model performance

#### Accuracy and bias

Overall the standard duration method shows the highest level of bias among the three methods—globally underestimating educational attainment values—as well as increased error relative to the two other models tested. Table 3 shows overall RMSE and median error for both average years of schooling and the standard deviation of education for all bin-splitting models. The standard duration model showed a median error of -0.47 years, which represents a substantial downward bias in predictions of mean attainment. We also observed an upward bias in estimates of the standard deviation of attainment, with a median error of 0.14 years. The standard duration method also had the highest average error in both average years of schooling, and the standard deviation of attainment.

**Table 3:** Predictive validity of tested models.

Model	RMSE in Mean	RMSE in SD	Median Error in Mean	Median Error in SD
Space-Time Distance	0.2601	0.2861	<0.0001	-0.0090
Nested Mixed Effects	0.4693	0.4987	-0.0207	0.1034
Standard Duration	0.9235	0.6113	-0.4683	0.1403

RMSE and median error in both mean education and the standard deviation of education, shown for each method assessed.

The space-time distance model produced the most accurate predictions, shown by the lowest RMSE, in both mean years of schooling (0.26 as compared to 0.92 for the standard duration method) and the standard deviation of educational attainment (0.28 compared to .6113 for the standard duration method), followed by the nested mixed effects model. The space-time distance model produced effectively unbiased estimates of both average attainment and the standard deviation of attainment. The nested mixed effects model predicted nearly unbiased estimates of mean attainment but showed a small upward bias in the prediction of the standard deviation of attainment, with a median error of 0.10.

#### Performance over number of bins

**Fig 1:**
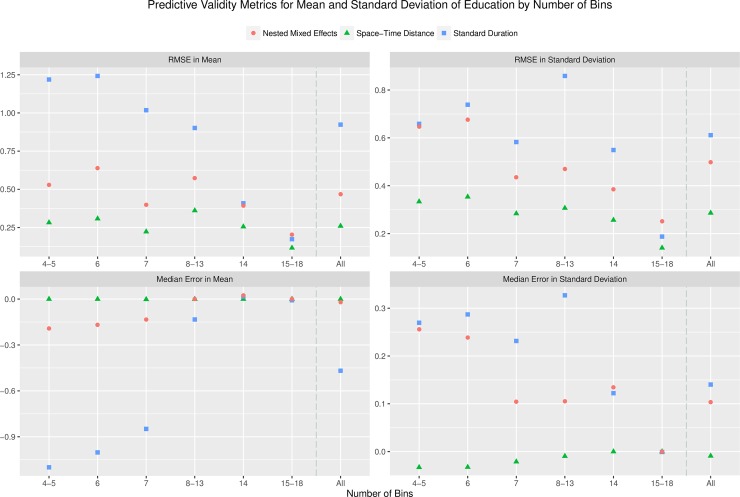
Predictive validity by number of bins. Predictive validity metrics for the mean and standard deviation of attainment are shown by number of bins present in the binning schema used. Space-time distance model results shown using hyper-parameter set with optimal RMSE in mean attainment. Overall the space-time distance model has the lowest error and bias, the nested mixed effects model performs intermediately, and the standard duration method has the poorest performance. All metrics of performance tend to improve as bin number increases, and the predictive task becomes easier.

Fig 1 shows the RMSE and median error in average years of schooling and the standard deviation of attainment across the number of bins in the binning schema applied. Generally, as the number of bins in the binning schema increased, all model showed increased accuracy. This is unsurprising as the difficultly of accurately predicting the proportion of individuals within each year rises directly with the width of the bins being split to single-year values. This effect was more pronounced for the standard duration model, which generally had the highest RMSE, and had markedly higher RMSE in mean attainment compared to both other models when the binning schema being split had fewer than 14 bins. The space-time distance model had the best performance in RMSE of mean attainment, ranging from an RMSE of 0.12 years for 15–18 bins to 0.32 years for 6 bins. As the number of bins decreased, both the space-time distance and nested mixed effect models showed slight increases in the RMSE of mean attainment. The space-time distance model had the most accurate predictions of the standard deviation of education across all numbers of bins, with an RMSE ranging from 0.14 for 15–18 bins to 0.36 for 6 bins, while the nested mixed effects model performed intermediately at higher numbers of bins and similarly to the standard duration method with fewer bins.

The space-time distance model produced nearly unbiased predictions of both the standard deviation and average years of education across all numbers of bins, with a highest absolute median error value of 0.001, as compared to 0.19 for the nested mixed effects model, and 1.10 for the standard duration method. The nested mixed effect model showed less biased predictions of mean attainment compared to the standard duration method, but both showed similar levels of upward bias in predictions of the standard deviation of attainment as the number of bins decreased, with a maximum median error for the standard duration method of 0.33 for 8–13 bins, and 0.26 for the nested mixed effects model at 4–5 bins. The standard duration method produced downward biased estimates of mean attainment and upward biased predictions of the standard deviation of attainment, with both phenomena inversely related to the number of bins being split.

#### Performance over true mean

**Fig 2:**
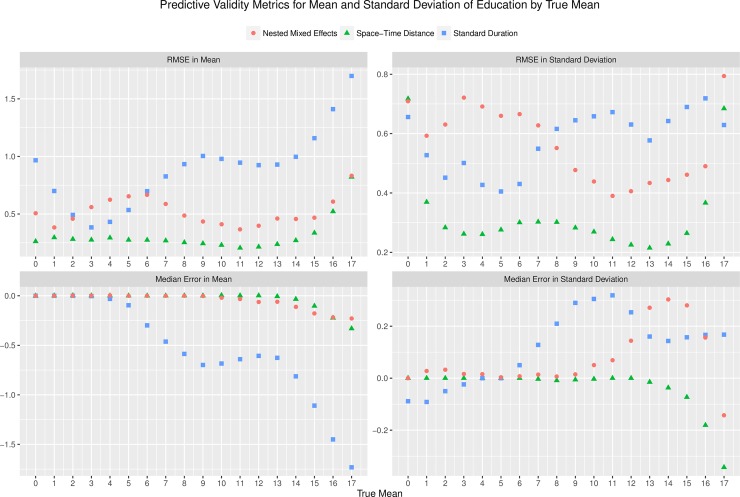
Predictive validity by true mean. Predictive validity metrics for the mean and standard deviation of attainment by one-year increments of true mean attainment in the population. The number shown indicates the lower bound of the interval, e.g. 15 indicates [15,16). Space-time distance model results shown using hyper-parameter set with optimal RMSE in mean attainment. Overall the order of model performance is similar to that shown in Fig 1, across the range of true mean values. The standard duration method increasingly underestimates educational attainment in highly educated populations.

Fig 2 shows the RMSE and median error in both average years and the standard deviation of education by the true mean attainment of the data being split in one-year increments. The RMSE of mean attainment estimates produced using the standard duration method increased as the true mean attainment of the population increased, up to a maximum of 1.68 for a true mean of 17–18 years. The median error of mean attainment from the standard duration method also became increasingly negative with higher true mean values, to a minimum value of -1.72 for a true mean of 17–18 years. Together these results show that the standard duration method underestimates mean attainment among more highly educated populations. The standard duration method also overestimates the standard deviation of attainment with higher true average attainment, although the RMSE in the standard deviation seems less clearly related to true average education.

The nested mixed effects model has poor accuracy in predicting the standard deviation of education, especially in lower true mean attainment populations. The model does perform better than the standard duration model in RMSE of mean attainment, although worse than the space-time distance model. It also generally produces unbiased predictions of both average years and the standard deviation of education, although it shows some increased bias in higher true mean populations. The space-time distance model performs the most accurately of the three models, showing the smallest RMSE in the mean and standard deviation of attainment across all levels of true mean attainment. For example, at a true mean attainment of 9–10 years, the RMSE in mean attainment for the space-time distance model is 0.24, compared to 0.43 for the nested mixed effects model and 1.00 for the standard duration method. The model shows almost no bias until higher true levels of attainment, where a slight increase in downward bias is present, as evidenced by a median error in mean attainment of -0.33 for a true mean of 17–18. The model has relatively stable RMSE in both the mean and the standard deviation of attainment across true mean attainment values.

### Space-time distance model hyper-parameter grid search

**Fig 3:**
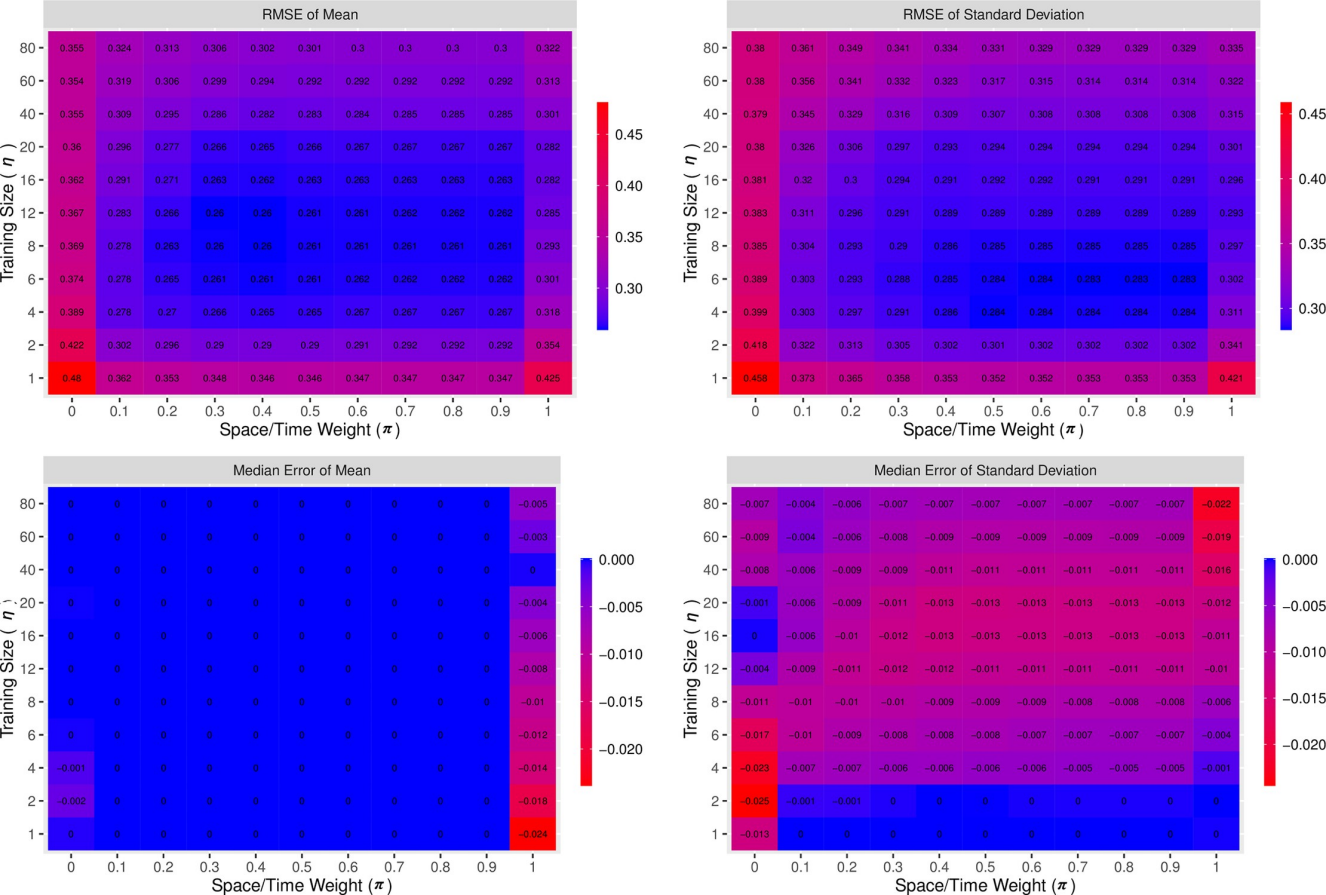
Hyper-parameter grid search predictive validity. Predictive validity metrics for the mean and standard deviation of attainment predictions of space-time distance model, over the grid of hyper-parameters tested using out-of-sample predictive validity. The grid search was remarkably planar in the middle of the search space, as the model does not seem to be that sensitive to hyper-parameter selection. The best hyper-parameter set with respect to RMSE in average attainment is a *π* of .6 and an *η* of 12, which was chosen at the hyper-parameter set to split the binned data.

Fig 3 shows the RMSE and median error in both average years and the standard deviation of education across the grid of hyper-parameters used in the space-time distance model that were tested using out-of-sample predictive validity. All outcome measures proved to be remarkably planar near their optima, with large areas of virtually identical RMSE and median error values near optimal parameter values. All predictive validity measures were also generally smooth with respect to hyper-parameters, and there were few local extrema.

The bias in the mean and standard deviation of attainment was close to zero across the vast majority of the grid of hyper-parameters, so optimal parameter values were chosen based on RMSE. The best hyper-parameter set with respect to RMSE in average attainment is a *π* of .6 and an *η* of 12. This parameter set also had very close to optimal predictive validity values in all other aspects tested, and was therefore chosen as the single best set for use in the model.

### Impact of standard duration method on global education trends

**Fig 4:**
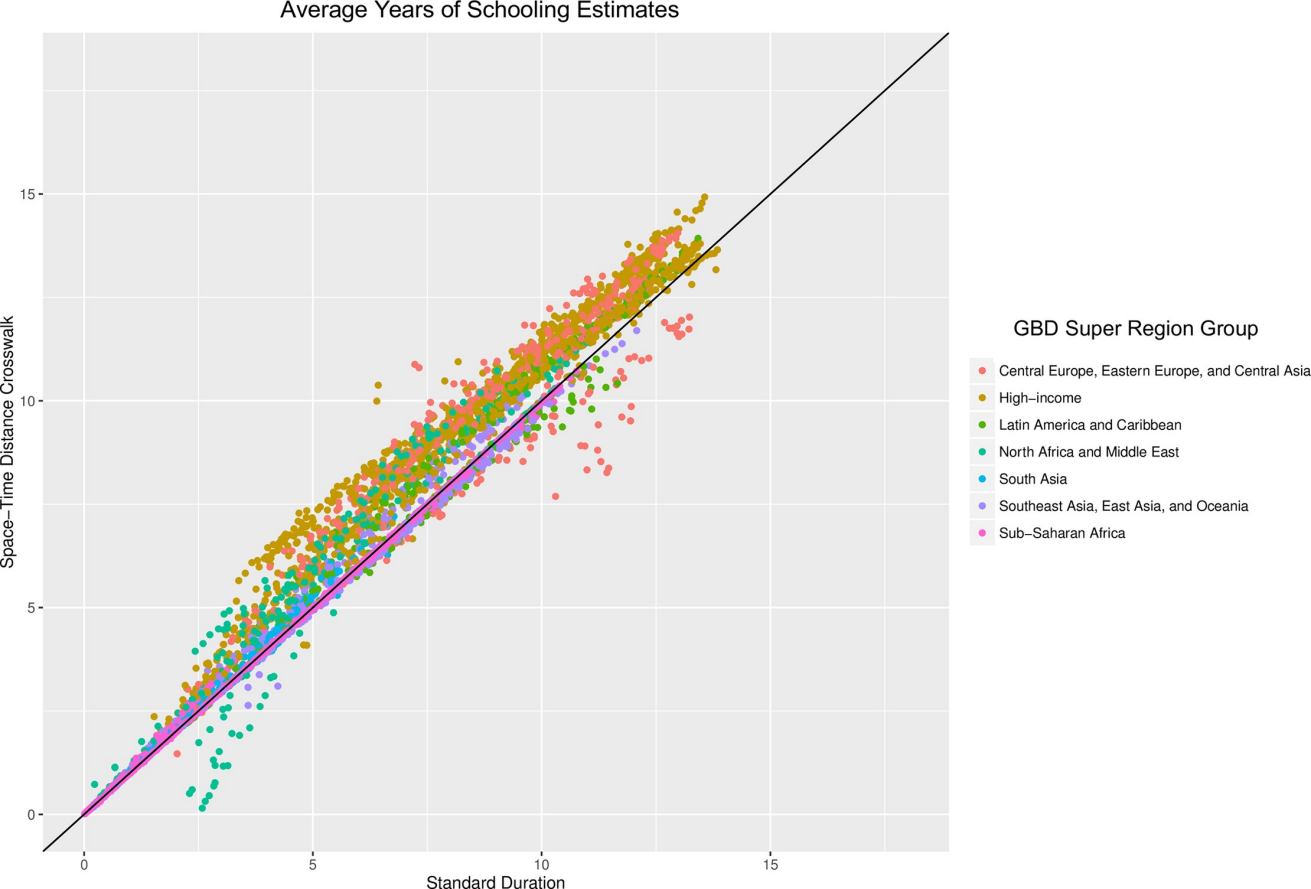
Impact of method choice on average years of schooling estimates. Average years of schooling estimated using the standard duration method (X axis), and the space-time distance model (Y axis) using best performing hyper-parameters. A line of equality added to show the point at which the two models produce identical estimates. Each point is a country-year-sex and five-year age group. Overall the predictions from the standard duration model are lower, with substantial differences between super-region grouping used in the GBD 2015 study, suggesting differential bias.

In order to explore the potential impact of the use of the standard duration model on global education trends, we used each model to produce average years of schooling estimates using our binned education data. Fig 4 shows a scatterplot of the average years of schooling estimated using both the standard duration method, and the space-time distance model, which was chosen due to better performance than the nested mixed effects model in out-of-sample validity testing. A line of equality shows the point at which the two models produce identical estimates for the sample country-year-sex and five-year age group. In general, similar trends are seen in the global education estimates to those observed in the out-of-sample predictive validity exercise. The standard duration model tends to produce lower estimates of average years of schooling, although this is not observed in all circumstances. There are also large differences in the deviations between the predictions in the two models, with some regions tending to have predictions further from the line of equality. This suggests that, as indicated by out-of-sample predictive validity testing, the standard duration method is biased to a differing degree depending on context, therefore affecting the comparability of results across different geographies and time periods.

## Conclusions

This study represents the first evaluation of the standard duration method for mapping binned education data onto single-year values and calculating average years of schooling. This method has been used widely by all major attempts to measure educational attainment, from the early estimates of Psacharopoulos and Arriagada to the more recent work by Barro and Lee and others. Average years of schooling estimates produced in this manner have been used widely, from being an essential component of the Human Development Index, to a key indicator for UNESCO and the World Bank, and a key measure of socio-economic status in numerous multi-country health studies. Nevertheless, as we show in this analysis, the standard duration method produces inaccurate and biased estimates of average years of schooling. This effect is uneven across the number of bins of attainment present in binning schemas and the level of true population mean years of schooling, which nearly assures that use of the method will produce less comparable results between geographies and time periods being measured. We show that the standard duration method is generally less accurate than models attempting to split bins into single-years and tends to substantially underestimate the average attainment of more highly educated populations. The use of this method has likely deflated global estimates of educational attainment, and induced inconsistencies between countries, in the most widely used education estimates at the time of publication. It is therefore clear that although the method is a very convenient approximation, it should only be used very cautiously, and comprehensive global estimates of educational attainment should employ a more nuanced approach.

This study demonstrates that an effective way to split bins of educational attainment into single-year values is to match the data source in question with a number of other surveys or census datasets that are close in terms of geography and time, and take the average of their school dropout pattern. While our analysis operationalizes this process with parameters, the specific number of surveys used and relative emphasis of space and time seems less to have a limited effect on the results. Therefore, the method could likely be replicated or possible even improved upon with expert opinion, especially when splitting a small number of surveys. Given the current widespread availability of single-year education datasets, most researchers should have no trouble finding suitable datasets to match with their binned education data sources.

Beyond improvements in accuracy and reductions in bias, the use of a space-time distance model represents a highly flexible approach to combining education data. The model allows for the use of any kind of binned and single-year data in concert. For many geographies it is only possible to find binned education data while for others only single-year data are available. The use of a space-time distance model allows for the combination of these data sources without concerns of compositional bias. Furthermore, the space-time distance approach produces highly unbiased single-year proportions with only minimal inaccuracy. Any lingering inaccuracy can be propagated through to final education estimates with the use of uncertainty intervals. The other advantage of the approach outlined in this analysis is that by using collapsed forms of person-level survey and census data to train the model, it is possible to split education bins for any custom age or sex groupings found in a given data source.

A main limitation of this study is that the results are representative only of the single-year training data and binning schemas used for out-of-sample predictive validity. There may be specific data sources that some researchers may wish to use that were not included in our analysis. Nevertheless, given that we have included 1,680 country-year datasets in this analysis—which to our knowledge represents the most comprehensive collection of education data to date—our results should robustly generalize to most geographies and time periods. Another factor for consideration in interpreting our results is that although we have characterized the variation in accuracy and bias over the principal dimensions that could affect model performance—the overall average level of education in the population and the number of bins present in the binning schema used—it is possible that other dimensions we are not aware of exist that should be considered in model selection. Similarly, although we have optimized several key hyper-parameters used in the proposed methods, other parameters and model combinations were not tested due to limits of computation burden. We hope that future studies may corroborate our findings and further refine the proposed methods. Furthermore, it is important to note that although average years of schooling is the most ubiquitously used measure of educational attainment, it does entail other sources of bias that may complicate its use [31,37–39]. We do not argue here that average years of schooling is the best metric of educational attainment, however in recognition of its prevalence in social science and health research, we recognize that evaluating and ameliorating bias in its estimation remains paramount.

Given the importance of educational attainment as a social determinant of health, indicator of socio-economic status, and metric of development, the accurate measurement of education is an important metrics aim. We have made available the code used in the analysis, and linked to a large number of data sources, so that other researchers may find it straightforward to implement a space-time distance model to produced unbiased and more accurate singe year education values. The best-performing method explored in this study is relatively easy to emulate, requiring only arithmetic and publicly available data from spatially and temporally proximate populations to implement. Therefore, we hope that its adoption in academic endeavors using measures of educational attainment can reduce bias, as well as improve the monitoring and scientific understanding of education.

## Supporting information

S1 File Detailed information on data sources. (DOCX)

S2 File Code for predictive validity exercise. (DOCX)

S1 Table Predictive validity by number of bins. Exact values of data shown in Fig 1. Predictive validity metrics for the mean and standard deviation of attainment by number of bins present in the binning schema used. Space-time distance model results shown using hyper-parameter set with optimal RMSE in mean attainment.
(DOCX)

S2 Table Predictive validity by true mean. Exact values of data shown in Fig 2. Predictive validity metrics for the mean and standard deviation of attainment by one-year increments of true mean attainment in the population. Space-time distance model results shown using hyper-parameter set with optimal RMSE in mean attainment.
(DOCX)

S3 Table Predictive validity of weighted regression space-time distance approach. (DOCX)

## References

[1] Millenium Development Goals Report 2015 [Internet]. New York: United Nations; 2015. Available: http://www.un.org/millenniumgoals/2015_MDG_Report/pdf/MDG%202015%20rev%20(July%201).pdf

[2] The Sustainable Development Goals Report 2016 [Internet]. New York: United Nations; 2016. Available: http://unstats.un.org/sdgs/report/2016/The%20Sustainable%20Development%20Goals%20Report%202016.pdf

[3] Basu AM, Stephenson R. Low levels of maternal education and the proximate determinants of childhood mortality: a little learning is not a dangerous thing. Soc Sci Med. 2005;60: 2011–2023. 10.1016/j.socscimed.2004.08.057 15743650 10.1016/j.socscimed.2004.08.057

[4] Bicego GT, Boerma JT. Maternal education and child survival: a comparative study of survey data from 17 countries. Soc Sci Med. 1993;36: 1207–1227. 8511650 10.1016/0277-9536(93)90241-u

[5] Cleland JG, Van Ginneken JK. Maternal education and child survival in developing countries: the search for pathways of influence. Soc Sci Med. 1988;27: 1357–1368. 3070762 10.1016/0277-9536(88)90201-8

[6] Desai S, Alva S. Maternal education and child health: is there a strong causal relationship? Demography. 1998;35: 71–81. 9512911

[7] Hobcraft J. Women’s education, child welfare and child survival: a review of the evidence. Health Transition Review. 1993;3: 159–175. 10146571

[8] Bhuiya A, Streatfield K. Mothers’ Education and Survival of Female Children in a Rural Area of Bangladesh. Population Studies. 1991;45: 253–264.

[9] Johri M, Subramanian SV, Koné GK, Dudeja S, Chandra D, Minoyan N, et al Maternal Health Literacy Is Associated with Early Childhood Nutritional Status in India. J Nutr. 2016; jn226290 10.3945/jn.115.226290 27306895 10.3945/jn.115.226290

[10] Makoka D, Masibo PK. Is there a threshold level of maternal education sufficient to reduce child undernutrition? Evidence from Malawi, Tanzania and Zimbabwe. BMC Pediatr. 2015;15 10.1186/s12887-015-0406-8 26297004 10.1186/s12887-015-0406-8 PMC4546212

[11] Hatt LE, Waters HR. Determinants of child morbidity in Latin America: a pooled analysis of interactions between parental education and economic status. Soc Sci Med. 2006;62: 375–386. 10.1016/j.socscimed.2005.06.007 16040175 10.1016/j.socscimed.2005.06.007

[12] Gakidou E, Cowling K, Lozano R, Murray CJ. Increased educational attainment and its effect on child mortality in 175 countries between 1970 and 2009: a systematic analysis. The Lancet. 2010;376: 959–974. 10.1016/S0140-6736(10)61257-3 10.1016/S0140-6736(10)61257-3 20851260

[13] Martin TC. Women’s Education and Fertility: Results from 26 Demographic and Health Surveys. Studies in Family Planning. 1995;26: 187–202. 10.2307/2137845 7482677

[14] Weinberger MB. The Relationship Between Women’s Education and Fertility: Selected Findings From the World Fertility Surveys. International Family Planning Perspectives. 1987;13: 35–46. 10.2307/2947826

[15] Winikoff B, Sullivan M. Assessing the role of family planning in reducing maternal mortality. Stud Fam Plann. 1987;18: 128–143. 3617120

[16] Fortney JA. The Importance of Family Planning in Reducing Maternal Mortality. Studies in Family Planning. 1987;18: 109–114. 10.2307/1966702 3590265

[17] MacNab YC. Bayesian multivariate disease mapping and ecological regression with errors in covariates: Bayesian estimation of DALYs and ‘preventable’ DALYs. Statist Med. 2009;28: 1369–1385. 10.1002/sim.3547 19206088 10.1002/sim.3547

[18] Land KC, Guralnik JM, Blazer DG. Estimating Increment-Decrement Life Tables with Multiple Covariates from Panel Data: The Case of Active Life Expectancy*. Demography. 31: 297–319. 10.2307/2061887 7926190

[19] Lozano R, Naghavi M, Foreman K, Lim S, Shibuya K, Aboyans V, et al Global and regional mortality from 235 causes of death for 20 age groups in 1990 and 2010: a systematic analysis for the Global Burden of Disease Study 2010. The Lancet. 2012;380: 2095–2128. 10.1016/S0140-6736(12)61728-0 10.1016/S0140-6736(12)61728-0 PMC10790329 23245604

[20] Lutz W. Education will be at the heart of 21st century demography. Vienna Yearbook of Population Research. 2010;8: 9–16.

[21] Global, regional, and national age–sex specific all-cause and cause-specific mortality for 240 causes of death, 1990–2013: a systematic analysis for the Global Burden of Disease Study 2013. The Lancet. 2015;385: 117–171. 10.1016/S0140-6736(14)61682-2 25530442 10.1016/S0140-6736(14)61682-2 PMC4340604

[22] Pope C III, Burnett RT, Thun MJ, et al Lung cancer, cardiopulmonary mortality, and long-term exposure to fine particulate air pollution. JAMA. 2002;287: 1132–1141. 10.1001/jama.287.9.1132 11879110 10.1001/jama.287.9.1132 PMC4037163

[23] Plummer M, Herrero R, Franceschi S, Meijer CJ, Snijders P, Bosch FX, et al Smoking and cervical cancer: pooled analysis of the IARC multi-centric case–control study. Cancer Causes & Control. 2003;14: 805–814. 14682438 10.1023/b:caco.0000003811.98261.3e

[24] Chen Z, Peto R, Zhou M, Iona A, Smith M, Yang L, et al Contrasting male and female trends in tobacco-attributed mortality in China: evidence from successive nationwide prospective cohort studies. The Lancet. 2015;386: 1447–1456. 10.1016/S0140-6736(15)00340-2 26466050 10.1016/S0140-6736(15)00340-2 PMC4691901

[25] Multiple Indicator Cluster Survey (MICS) | Statistics and Monitoring | UNICEF [Internet]. [cited 23 Aug 2016]. Available: http://www.unicef.org/statistics/index_24302.html

[26] The DHS Program—Data [Internet]. [cited 23 Aug 2016]. Available: http://dhsprogram.com/data/

[27] IPUMS International [Internet]. [cited 23 Aug 2016]. Available: https://international.ipums.org/international/

[28] Psacharopoulos G, Arriagada A-M. The Educational Attainment of the Labor Force: An International Comparison [Internet]. World Bank; 1986 10 Available: http://documents.worldbank.org/curated/en/663961468135311923/pdf/edt38.pdf

[29] Barro RJ, Lee JW. A new data set of educational attainment in the world, 1950–2010. Journal of Development Economics. 2013;104: 184–198. 10.1016/j.jdeveco.2012.10.001

[30] Barro RJ, Lee J-W. International comparisons of educational attainment. Journal of monetary economics. 1993;32: 363–394.

[31] Barro RJ, Lee JW. International Measures of Schooling Years and Schooling Quality. The American Economic Review. 1996;86: 218–223.

[32] Barro RJ, Lee J-W. Sources of economic growth Carnegie-Rochester conference series on public policy. Elsevier; 1994 pp. 1–46. Available: http://www.sciencedirect.com/science/article/pii/0167223194900027

[33] Nehru V, Swanson E, Dubey A. A new database on human capital stock in developing and industrial countries: Sources, methodology, and results. Journal of Development Economics. 1995;46: 379–401. 10.1016/0304-3878(94)00054-G

[34] de la Fuente A, Doménech R. Educational attainment in the OECD, 1960–2010. Updated series and a comparison with other sources. Economics of Education Review. 2015;48: 56–74. 10.1016/j.econedurev.2015.05.004

[35] De la Fuente Á, Doménech R. Educational Attainment in the OECD, 1960–95. 2002; Available: http://papers.ssrn.com/sol3/papers.cfm?abstract_id=317689

[36] de la Fuente A, Doménech R. Human Capital in Growth Regressions: How Much Difference Does Data Quality Make? Journal of the European Economic Association. 2006;4: 1–36.

[37] Battistin E, De Nadai M, Sianesi B. Misreported schooling, multiple measures and returns to educational qualifications. Journal of Econometrics. 2014;181: 136–150. 10.1016/j.jeconom.2014.03.002

[38] Hu Y, Xiao R, Zhong X. Misclassification of Schooling in Survey and Transcript Samples.: 19.

[39] Barro RJ, Lee J-W. Educational Attainment—Quantity and Quality of Schooling [Internet] Oxford University Press; 2015 Available: http://www.oxfordscholarship.com/view/10.1093/acprof:oso/9780199379231.001.0001/acprof-9780199379231-chapter-6

[40] IPUMS-I: descr: YRSCHOOL [Internet]. [cited 25 Aug 2016]. Available: https://international.ipums.org/international-action/variables/YRSCHOOL#comparability_section

[41] Hoeting JA, Madigan D, Raftery AE, Volinsky CT. Bayesian model averaging: a tutorial (with comments by M. Clyde, David Draper and E. I. George, and a rejoinder by the authors. Statist Sci. 1999;14: 382–417. 10.1214/ss/1009212519

[42] King G. “Truth” Is Stranger than Prediction, More Questionable than Causal Inference. American Journal of Political Science. 1991;35: 1047–1053. 10.2307/2111506

[43] Stone M. Cross-validatory choice and assessment of statistical predictions. Journal of the Royal Statistical Society Series B (Methodological). 1974; 111–147.

[44] Power M. Theoretical Modelling AspectsThe predictive validation of ecological and environmental models. Ecological Modelling. 1993;68: 33–50. 10.1016/0304-3800(93)90106-3

[45] Tashman LJ. Out-of-sample tests of forecasting accuracy: an analysis and review. International Journal of Forecasting. 2000;16: 437–450. 10.1016/S0169-2070(00)00065-0

[46] Foreman KJ, Lozano R, Lopez AD, Murray CJ. Modeling causes of death: an integrated approach using CODEm. Population Health Metrics. 2012;10: 1 10.1186/1478-7954-10-1 22226226 10.1186/1478-7954-10-1 PMC3315398

[47] Kuhn M, Johnson K. Applied Predictive Modeling [Internet] New York, NY: Springer New York; 2013 Available: http://link.springer.com/10.1007/978-1-4614-6849-3

[48] Thomas V, Wang Y, Fan X. Measuring education inequality: Gini coefficients of education [Internet] World Bank Publications; 2001 Available: http://books.google.com/books?hl=en&lr=&id=cVkVi5bzEQkC&oi=fnd&pg=PA3&dq=%22deviations+and+Gini+coefficients+are+often+chosen+as+measures+of+inequality.%22+%22Gini+coefficients+in+measuring+education+inequality.+They+estimated+the+Gini+coefficient+based%22+%22of+school+attainment+were+used+in+a+few+studies.+Only+four+previous+studies+wer&ots=zOW2V3Gf2j&sig=E8wdxkenwq6f9QoIJxKNxfluRRk

[49] Inter-American Development Bank, editor. Facing up to inequality in Latin America Washington, DC: Johns Hopkins Univ. Press; 1998.

[50] Birdsall N, Londono JL. Asset Inequality Matters: An Assessment of the World Bank’s Approach to Poverty Reduction. The American Economic Review. 1997;87: 32–37.

