# Supplementary material for: Improving the estimation of educational attainment: New methods for assessing average years of schooling from binned data
Source: PLoS One. 2018 Nov 29;13(11):e0208019. doi: 10.1371/journal.pone.0208019 (PMC6264843; doi:10.1371/journal.pone.0208019)
Supplement: S1 File — (DOCX) [file pone.0208019.s001.docx]

**S1 File. Detailed Information on Data Sources.**

For meta-data regarding each source, and information regarding data availability, see the Global Health Data Exchange website: <http://ghdx.healthdata.org>

In the below tables, the following data family abbreviations are used to save space:

DHS = Demographic and Health Survey

WHS = World Health Survey

EUR = Eurobarometer Survey

ISSP = Integrated Social Survey Programme

IPUMS = Integrated Public Use Microdata Samples

**Table A. Single-Year Data Sources**

| **ISO3 Country Code** | **Sources Used** |
| --- | --- |
| ALB | 2008 DHS,2014 EUR,2015 EUR |
| ARE | 2003 WHS |
| ARG | 1970 IPUMS,1980 IPUMS,1991 IPUMS,2007 ISSP,2009 ISSP,2010 IPUMS,2010 ISSP,2012 ISSP |
| ARM | 2000 DHS,2005 DHS,2010 DHS |
| AUS | 1990 ISSP,1991 ISSP,1992 ISSP,1993 ISSP,1994 ISSP,1995 ISSP,1996 ISSP,1998 ISSP,1999 ISSP,2001 ISSP,2002 ISSP,2003 ISSP,2003 WHS,2004 ISSP,2005 ISSP,2006 ISSP,2007 ISSP,2008 ISSP,2009 ISSP,2011 ISSP,2012 ISSP |
| AUT | 1989 ISSP,1994 ISSP,1995 EUR,1995 ISSP,1996 EUR,1997 EUR,1998 EUR,1998 ISSP,1999 EUR,1999 ISSP,2000 EUR,2000 ISSP,2001 EUR,2001 ISSP,2002 EUR,2003 EUR,2003 ISSP,2004 EUR,2004 ISSP,2005 EUR,2006 EUR,2007 EUR,2008 EUR,2009 EUR,2010 EUR,2011 EUR,2012 EUR,2013 EUR,2014 EUR,2015 EUR |
| AZE | 2006 DHS |
| BDI | 2010 DHS |
| BEL | 1990 EUR,1992 EUR,1993 EUR,1994 EUR,1995 EUR,1996 EUR,1997 EUR,1998 EUR,1999 EUR,2000 EUR,2001 EUR,2002 EUR,2003 EUR,2004 EUR,2005 EUR,2006 EUR,2007 EUR,2008 EUR,2008 ISSP,2009 EUR,2009 ISSP,2010 EUR,2010 ISSP,2011 EUR,2011 ISSP,2012 EUR,2013 EUR,2014 EUR,2015 EUR |
| BEN | 1996 DHS,2001 DHS,2006 DHS,2011 DHS |
| BFA | 1985 IPUMS,1992 DHS,1998 DHS,2003 DHS,2003 WHS,2010 DHS |
| BGD | 1993 DHS,1996 DHS,1997 ISSP,1999 DHS,2003 WHS,2004 DHS,2007 DHS,2011 DHS |
| BGR | 1992 ISSP,1993 ISSP,1996 ISSP,1997 ISSP,1998 ISSP,1999 ISSP,2000 ISSP,2002 ISSP,2003 ISSP,2004 EUR,2004 ISSP,2005 EUR,2005 ISSP,2006 EUR,2007 EUR,2007 ISSP,2008 EUR,2009 EUR,2009 ISSP,2010 EUR,2010 ISSP,2011 EUR,2011 ISSP,2012 EUR,2012 ISSP,2013 EUR,2014 EUR,2015 EUR |
| BIH | 2003 WHS |
| BOL | 1976 IPUMS,1992 IPUMS,1993 DHS,1998 DHS,2003 DHS,2008 DHS |
| BRA | 1991 DHS,1996 DHS,2001 ISSP,2002 ISSP,2003 WHS,2004 ISSP |
| CAF | 1994 DHS |
| CAN | 1995 ISSP,1996 ISSP,1997 ISSP,1998 ISSP,1999 ISSP,2000 ISSP,2001 ISSP,2003 ISSP,2004 ISSP,2005 ISSP,2006 ISSP,2010 ISSP,2012 ISSP |
| CHE | 1996 ISSP,1997 ISSP,1998 ISSP,2002 ISSP,2003 ISSP,2004 ISSP,2005 EUR,2005 ISSP,2006 ISSP,2007 ISSP,2008 ISSP,2009 ISSP,2010 EUR,2010 ISSP,2011 ISSP,2012 ISSP |
| CHL | 1960 IPUMS,1970 IPUMS,1982 IPUMS,1992 IPUMS,1998 ISSP,1999 ISSP,2000 ISSP,2001 ISSP,2002 IPUMS,2002 ISSP,2003 ISSP,2004 ISSP,2006 ISSP,2007 ISSP,2008 ISSP,2009 ISSP,2010 ISSP,2011 ISSP,2012 ISSP |
| CHN | 2002 WHS,2009 ISSP,2011 ISSP,2012 ISSP |
| CIV | 1994 DHS,1998 DHS,2003 WHS,2011 DHS |
| CMR | 1976 IPUMS,1987 IPUMS,1991 DHS,1998 DHS,2004 DHS,2005 IPUMS,2011 DHS |
| COD | 2007 DHS,2013 DHS |
| COG | 2003 WHS,2005 DHS,2011 DHS |
| COL | 1964 IPUMS,1973 IPUMS,1985 IPUMS,1990 DHS,1993 IPUMS,1995 DHS,2000 DHS,2004 DHS,2009 DHS |
| COM | 1996 DHS,2003 WHS,2012 DHS |
| CRI | 1963 IPUMS,1973 IPUMS,1984 IPUMS,2000 IPUMS,2011 IPUMS |
| CYP | 1996 ISSP,1997 ISSP,1998 ISSP,1999 ISSP,2001 ISSP,2002 ISSP,2004 EUR,2004 ISSP,2005 EUR,2005 ISSP,2006 EUR,2007 EUR,2007 ISSP,2008 EUR,2008 ISSP,2009 EUR,2009 ISSP,2010 EUR,2011 EUR,2012 EUR,2013 EUR,2014 EUR,2015 EUR |
| CZE | 1992 ISSP,1993 ISSP,1994 ISSP,1995 ISSP,1996 ISSP,1997 ISSP,1998 ISSP,1999 ISSP,2000 ISSP,2001 ISSP,2002 EUR,2002 ISSP,2003 ISSP,2003 WHS,2004 EUR,2004 ISSP,2005 EUR,2005 ISSP,2006 EUR,2006 ISSP,2007 EUR,2007 ISSP,2008 EUR,2008 ISSP,2009 EUR,2009 ISSP,2010 EUR,2010 ISSP,2011 EUR,2011 ISSP,2012 EUR,2012 ISSP,2013 EUR,2014 EUR,2015 EUR |
| DEU | 1989 ISSP,1990 EUR,1990 ISSP,1991 ISSP,1992 EUR,1992 ISSP,1993 EUR,1993 ISSP,1994 EUR,1995 EUR,1995 ISSP,1996 EUR,1997 EUR,1998 EUR,1998 ISSP,1999 EUR,1999 ISSP,2000 EUR,2000 ISSP,2001 EUR,2001 ISSP,2002 EUR,2002 ISSP,2003 EUR,2003 ISSP,2004 EUR,2004 ISSP,2005 EUR,2005 ISSP,2006 EUR,2006 ISSP,2007 EUR,2007 ISSP,2008 EUR,2008 ISSP,2009 EUR,2009 ISSP,2010 EUR,2010 ISSP,2011 EUR,2011 ISSP,2012 EUR,2012 ISSP,2013 EUR,2014 EUR,2015 EUR |
| DMA | 2005 ISSP,2006 ISSP,2007 ISSP,2008 ISSP |
| DNK | 1990 EUR,1992 EUR,1993 EUR,1994 EUR,1995 EUR,1996 EUR,1997 EUR,1997 ISSP,1998 EUR,1998 ISSP,1999 EUR,2000 EUR,2000 ISSP,2001 EUR,2001 ISSP,2002 EUR,2002 ISSP,2003 EUR,2003 ISSP,2004 EUR,2004 ISSP,2005 EUR,2005 ISSP,2006 EUR,2006 ISSP,2007 EUR,2008 EUR,2008 ISSP,2009 EUR,2009 ISSP,2010 EUR,2010 ISSP,2011 EUR,2011 ISSP,2012 EUR,2012 ISSP,2013 EUR,2014 EUR,2015 EUR |
| DOM | 1960 IPUMS,1970 IPUMS,1981 IPUMS,1991 DHS,1996 DHS,1999 DHS,2002 DHS,2002 IPUMS,2003 WHS,2007 DHS,2010 IPUMS,2013 DHS |
| ECU | 1962 IPUMS,1974 IPUMS,1982 IPUMS,1990 IPUMS,2001 IPUMS,2003 WHS,2010 IPUMS |
| EGY | 1992 DHS,1995 DHS,2000 DHS,2005 DHS,2008 DHS,2014 DHS |
| ERI | 1995 DHS,2002 DHS |
| ESP | 1990 EUR,1992 EUR,1993 EUR,1993 ISSP,1994 EUR,1995 EUR,1995 ISSP,1996 EUR,1997 EUR,1997 ISSP,1998 EUR,1998 ISSP,1999 EUR,1999 ISSP,2000 EUR,2000 ISSP,2001 EUR,2001 ISSP,2002 EUR,2002 ISSP,2003 EUR,2003 ISSP,2003 WHS,2004 EUR,2004 ISSP,2005 EUR,2005 ISSP,2006 EUR,2006 ISSP,2007 EUR,2008 EUR,2008 ISSP,2009 EUR,2009 ISSP,2010 EUR,2010 ISSP,2011 EUR,2011 ISSP,2012 EUR,2012 ISSP,2013 EUR,2014 EUR,2015 EUR |
| EST | 2003 WHS,2004 EUR,2005 EUR,2006 EUR,2007 EUR,2008 EUR,2009 EUR,2009 ISSP,2010 EUR,2011 EUR,2012 EUR,2013 EUR,2014 EUR,2015 EUR |
| ETH | 2000 DHS,2003 WHS,2005 DHS,2010 DHS |
| FIN | 1995 EUR,1996 EUR,1997 EUR,1998 EUR,1999 EUR,2000 EUR,2000 ISSP,2001 EUR,2001 ISSP,2002 EUR,2002 ISSP,2003 EUR,2003 ISSP,2004 EUR,2004 ISSP,2004 WHS,2005 EUR,2005 ISSP,2006 EUR,2006 ISSP,2007 EUR,2007 ISSP,2008 EUR,2008 ISSP,2009 EUR,2009 ISSP,2010 EUR,2010 ISSP,2011 EUR,2011 ISSP,2012 EUR,2012 ISSP,2013 EUR,2014 EUR,2015 EUR |
| FRA | 1990 EUR,1992 EUR,1993 EUR,1994 EUR,1995 EUR,1996 EUR,1996 ISSP,1997 EUR,1997 ISSP,1998 EUR,1998 ISSP,1999 EUR,1999 ISSP,2000 EUR,2001 EUR,2001 ISSP,2002 EUR,2002 ISSP,2003 EUR,2003 ISSP,2003 WHS,2004 EUR,2004 ISSP,2005 EUR,2005 ISSP,2006 EUR,2006 ISSP,2007 EUR,2007 ISSP,2008 EUR,2008 ISSP,2009 EUR,2009 ISSP,2010 EUR,2010 ISSP,2011 EUR,2011 ISSP,2012 EUR,2012 ISSP,2013 EUR,2014 EUR,2015 EUR |
| GAB | 2000 DHS,2012 DHS |
| GBR | 1994 ISSP,1998 ISSP,1999 ISSP,2000 ISSP,2001 ISSP,2002 ISSP,2003 ISSP,2004 ISSP,2005 ISSP,2006 ISSP,2007 ISSP,2008 ISSP,2009 ISSP,2010 ISSP,2011 ISSP,2012 ISSP |
| GEO | 2003 WHS |
| GHA | 1984 IPUMS,1993 DHS,1998 DHS,2000 IPUMS,2003 DHS,2003 WHS,2008 DHS,2010 IPUMS,2014 DHS |
| GIN | 1983 IPUMS,1996 IPUMS,1999 DHS,2005 DHS,2012 DHS |
| GMB | 2013 DHS |
| GRC | 1990 EUR,1992 EUR,1993 EUR,1994 EUR,1995 EUR,1996 EUR,1997 EUR,1998 EUR,1999 EUR,2000 EUR,2001 EUR,2002 EUR,2003 EUR,2004 EUR,2005 EUR,2006 EUR,2007 EUR,2008 EUR,2009 EUR,2010 EUR,2011 EUR,2012 EUR,2013 EUR,2014 EUR,2015 EUR |
| GTM | 1995 DHS |
| GUY | 2009 DHS |
| HND | 2005 DHS,2011 DHS |
| HRV | 2003 WHS,2004 EUR,2005 EUR,2006 EUR,2006 ISSP,2007 EUR,2007 ISSP,2008 EUR,2008 ISSP,2009 EUR,2009 ISSP,2010 EUR,2010 ISSP,2011 EUR,2011 ISSP,2012 EUR,2012 ISSP,2013 EUR,2014 EUR,2015 EUR |
| HTI | 1982 IPUMS,1994 DHS,2000 DHS,2003 IPUMS,2005 DHS,2012 DHS |
| HUN | 1989 ISSP,1990 ISSP,1992 ISSP,1993 ISSP,1994 ISSP,1995 ISSP,1996 ISSP,1999 ISSP,2002 EUR,2003 WHS,2004 EUR,2004 ISSP,2005 EUR,2005 ISSP,2006 EUR,2006 ISSP,2007 EUR,2008 EUR,2008 ISSP,2009 EUR,2009 ISSP,2010 EUR,2011 EUR,2012 EUR,2013 EUR,2014 EUR,2015 EUR |
| IDN | 1991 DHS,1994 DHS,1997 DHS,2002 DHS,2007 DHS,2012 DHS |
| IND | 1983 IPUMS,1992 DHS,1998 DHS,2003 WHS,2005 DHS,2012 ISSP |
| IRL | 1989 ISSP,1990 EUR,1992 EUR,1993 EUR,1993 ISSP,1994 EUR,1995 EUR,1995 ISSP,1996 EUR,1996 ISSP,1997 EUR,1998 EUR,1998 ISSP,1999 EUR,2000 EUR,2000 ISSP,2001 EUR,2002 EUR,2002 ISSP,2003 EUR,2003 ISSP,2003 WHS,2004 EUR,2004 ISSP,2005 EUR,2005 ISSP,2006 EUR,2006 ISSP,2007 EUR,2007 ISSP,2008 EUR,2008 ISSP,2009 EUR,2010 EUR,2011 EUR,2012 EUR,2012 ISSP,2013 EUR,2014 EUR,2015 EUR |
| ISL | 2003 EUR,2005 EUR,2009 ISSP,2010 EUR,2011 EUR,2012 EUR,2012 ISSP,2013 EUR,2014 EUR |
| ISR | 1989 ISSP,1990 ISSP,1991 ISSP,1993 ISSP,1994 ISSP,1996 ISSP,1997 ISSP,1998 ISSP,1999 ISSP,2000 ISSP,2001 ISSP,2002 ISSP,2003 ISSP,2003 WHS,2004 ISSP,2005 ISSP,2006 ISSP,2007 ISSP,2008 ISSP,2009 ISSP,2010 ISSP,2011 ISSP,2012 ISSP |
| ITA | 1989 ISSP,1990 EUR,1990 ISSP,1991 ISSP,1992 EUR,1993 EUR,1993 ISSP,1994 EUR,1994 ISSP,1995 EUR,1995 ISSP,1996 EUR,1996 ISSP,1997 EUR,1997 ISSP,1998 EUR,1998 ISSP,1999 EUR,2000 EUR,2001 EUR,2001 ISSP,2002 EUR,2003 EUR,2004 EUR,2005 EUR,2006 EUR,2007 EUR,2008 EUR,2008 ISSP,2009 EUR,2009 ISSP,2010 EUR,2011 EUR,2011 ISSP,2012 EUR,2013 EUR,2014 EUR,2015 EUR |
| JAM | 1991 IPUMS,2001 IPUMS |
| JOR | 1990 DHS,1997 DHS,2002 DHS,2007 DHS,2012 DHS |
| JPN | 1993 ISSP,1994 ISSP,1995 ISSP,1996 ISSP,1997 ISSP,1998 ISSP,1999 ISSP,2000 ISSP,2001 ISSP,2002 ISSP,2003 ISSP,2004 ISSP,2005 ISSP,2006 ISSP,2007 ISSP,2008 ISSP,2009 ISSP,2010 ISSP,2011 ISSP,2012 ISSP |
| KAZ | 1995 DHS,1999 DHS,2003 WHS |
| KEN | 1993 DHS,1998 DHS,2003 DHS,2004 WHS,2008 DHS,2014 DHS |
| KGZ | 1997 DHS,2012 DHS |
| KHM | 2000 DHS,2005 DHS,2010 DHS,2014 DHS |
| KOR | 2003 ISSP,2004 ISSP,2005 ISSP,2006 ISSP,2007 ISSP,2008 ISSP,2009 ISSP,2010 ISSP,2011 ISSP,2012 ISSP |
| LAO | 2003 WHS |
| LBR | 1974 IPUMS,2006 DHS,2013 DHS |
| LKA | 2003 WHS |
| LSO | 2004 DHS,2009 DHS |
| LTU | 2004 EUR,2005 EUR,2006 EUR,2007 EUR,2008 EUR,2009 EUR,2010 EUR,2010 ISSP,2011 EUR,2011 ISSP,2012 EUR,2012 ISSP,2013 EUR,2014 EUR,2015 EUR |
| LUX | 1992 EUR,1993 EUR,1994 EUR,1995 EUR,1996 EUR,1997 EUR,1998 EUR,1999 EUR,2000 EUR,2001 EUR,2002 EUR,2003 EUR,2003 WHS,2004 EUR,2005 EUR,2006 EUR,2007 EUR,2008 EUR,2009 EUR,2010 EUR,2011 EUR,2012 EUR,2013 EUR,2014 EUR,2015 EUR |
| LVA | 1995 ISSP,1996 ISSP,1998 ISSP,1999 ISSP,2000 ISSP,2001 ISSP,2002 ISSP,2003 ISSP,2003 WHS,2004 EUR,2004 ISSP,2005 EUR,2005 ISSP,2006 EUR,2006 ISSP,2007 EUR,2007 ISSP,2008 EUR,2008 ISSP,2009 EUR,2009 ISSP,2010 EUR,2010 ISSP,2011 EUR,2012 EUR,2012 ISSP,2013 EUR,2014 EUR,2015 EUR |
| MAR | 1982 IPUMS,1992 DHS,1994 IPUMS,2003 DHS,2004 IPUMS |
| MDA | 2005 DHS |
| MDG | 1992 DHS,1997 DHS,2003 DHS,2008 DHS |
| MDV | 2009 DHS |
| MEX | 1960 IPUMS,1970 IPUMS,1990 IPUMS,1995 IPUMS,2000 IPUMS,2000 ISSP,2002 ISSP,2003 WHS,2004 ISSP,2005 IPUMS,2005 ISSP,2007 ISSP,2008 ISSP,2010 IPUMS,2010 ISSP,2012 ISSP |
| MKD | 2007 EUR,2008 EUR,2009 EUR,2010 EUR,2011 EUR,2012 EUR,2013 EUR,2014 EUR,2015 EUR |
| MLI | 1995 DHS,1998 IPUMS,2001 DHS,2003 WHS,2006 DHS,2009 IPUMS,2012 DHS |
| MLT | 2004 EUR,2005 EUR,2006 EUR,2007 EUR,2008 EUR,2009 EUR,2010 EUR,2011 EUR,2012 EUR,2013 EUR,2014 EUR,2015 EUR |
| MMR | 2003 WHS |
| MNE | 2011 EUR,2012 EUR,2013 EUR,2014 EUR,2015 EUR |
| MOZ | 1997 DHS,2003 DHS,2011 DHS |
| MRT | 2000 DHS,2003 WHS |
| MUS | 2003 WHS |
| MWI | 1987 IPUMS,1992 DHS,1998 IPUMS,2000 DHS,2003 WHS,2004 DHS,2008 IPUMS,2010 DHS |
| MYS | 2003 WHS |
| NAM | 1992 DHS,2000 DHS,2003 WHS,2006 DHS,2013 DHS |
| NER | 1992 DHS,1998 DHS,2006 DHS,2012 DHS |
| NGA | 2003 DHS,2008 DHS,2013 DHS |
| NIC | 1971 IPUMS,1995 IPUMS,1997 DHS,2001 DHS,2005 IPUMS |
| NLD | 1989 ISSP,1990 EUR,1991 ISSP,1992 EUR,1993 EUR,1993 ISSP,1994 EUR,1994 ISSP,1995 EUR,1995 ISSP,1996 EUR,1997 EUR,1997 ISSP,1998 EUR,1998 ISSP,1999 EUR,2000 EUR,2000 ISSP,2001 EUR,2002 EUR,2002 ISSP,2003 EUR,2003 ISSP,2004 EUR,2004 ISSP,2005 EUR,2005 ISSP,2006 EUR,2006 ISSP,2007 EUR,2008 EUR,2008 ISSP,2009 EUR,2010 EUR,2011 EUR,2011 ISSP,2012 EUR,2013 EUR,2014 EUR,2015 EUR |
| NOR | 1989 ISSP,1990 EUR,1991 EUR,1991 ISSP,1992 EUR,1992 ISSP,1993 EUR,1993 ISSP,1994 EUR,1994 ISSP,1995 EUR,1995 ISSP,1996 EUR,1996 ISSP,1997 ISSP,1998 ISSP,1999 EUR,1999 ISSP,2000 EUR,2000 ISSP,2001 EUR,2001 ISSP,2002 ISSP,2003 EUR,2003 ISSP,2003 WHS,2004 ISSP,2005 EUR,2005 ISSP,2006 ISSP,2007 ISSP,2008 ISSP,2009 ISSP,2010 EUR,2010 ISSP,2011 EUR,2011 ISSP,2012 ISSP |
| NPL | 1996 DHS,2001 DHS,2003 WHS,2006 DHS,2011 DHS |
| NZL | 1991 ISSP,1992 ISSP,1993 ISSP,1994 ISSP,1995 ISSP,1996 ISSP,1997 ISSP |
| PAK | 1990 DHS,2003 WHS,2006 DHS,2012 DHS |
| PAN | 1960 IPUMS,1970 IPUMS,1980 IPUMS,1990 IPUMS,2000 IPUMS,2010 IPUMS |
| PER | 1991 DHS,1996 DHS,2000 DHS,2003 DHS,2004 DHS,2005 DHS,2006 DHS,2007 DHS,2008 DHS,2009 DHS,2010 DHS,2011 DHS,2012 DHS,2013 DHS,2014 DHS |
| PHL | 1991 ISSP,1992 ISSP,1993 DHS,1993 ISSP,1994 ISSP,1995 ISSP,1996 ISSP,1997 ISSP,1998 DHS,1998 ISSP,1999 ISSP,2000 ISSP,2001 ISSP,2002 ISSP,2003 DHS,2003 ISSP,2003 WHS,2004 ISSP,2005 ISSP,2006 ISSP,2007 ISSP,2008 DHS,2008 ISSP,2009 ISSP,2010 ISSP,2011 ISSP,2012 ISSP,2013 DHS |
| POL | 1991 ISSP,1992 ISSP,1993 ISSP,1996 ISSP,1997 ISSP,1998 ISSP,1999 ISSP,2001 ISSP,2002 EUR,2002 ISSP,2003 ISSP,2004 EUR,2004 ISSP,2005 EUR,2006 EUR,2006 ISSP,2007 EUR,2007 ISSP,2008 EUR,2008 ISSP,2009 EUR,2009 ISSP,2010 EUR,2011 EUR,2011 ISSP,2012 EUR,2012 ISSP,2013 EUR,2014 EUR,2015 EUR |
| PRI | 1970 IPUMS,1980 IPUMS,2010 IPUMS |
| PRT | 1990 EUR,1992 EUR,1993 EUR,1994 EUR,1995 EUR,1996 EUR,1997 EUR,1997 ISSP,1998 EUR,1998 ISSP,1999 EUR,1999 ISSP,2000 EUR,2000 ISSP,2001 EUR,2002 EUR,2002 ISSP,2003 EUR,2003 ISSP,2003 WHS,2004 EUR,2004 ISSP,2005 EUR,2005 ISSP,2006 EUR,2006 ISSP,2007 EUR,2008 EUR,2008 ISSP,2009 EUR,2009 ISSP,2010 EUR,2011 EUR,2011 ISSP,2012 EUR,2013 EUR,2014 EUR,2015 EUR |
| PRY | 1962 IPUMS,1972 IPUMS,1982 IPUMS,1990 DHS,1992 IPUMS,2002 IPUMS,2003 WHS |
| ROU | 2004 EUR,2005 EUR,2006 EUR,2007 EUR,2008 EUR,2009 EUR,2010 EUR,2011 EUR,2012 EUR,2013 EUR,2014 EUR,2015 EUR |
| RUS | 1991 ISSP,1992 ISSP,1993 ISSP,1994 ISSP,1995 ISSP,1996 ISSP,1997 ISSP,1998 ISSP,1999 ISSP,2000 ISSP,2001 ISSP,2002 ISSP,2003 ISSP,2003 WHS,2004 ISSP,2005 ISSP,2006 ISSP,2007 ISSP,2008 ISSP,2009 ISSP,2010 ISSP,2011 ISSP,2012 ISSP |
| RWA | 1992 DHS,2000 DHS,2002 IPUMS,2005 DHS,2010 DHS |
| SEN | 1988 IPUMS,1992 DHS,2002 IPUMS,2003 WHS,2005 DHS,2010 DHS,2012 DHS,2014 DHS |
| SLE | 2008 DHS,2013 DHS |
| SLV | 1992 IPUMS,2007 IPUMS |
| SRB | 2012 EUR,2013 EUR,2014 EUR,2015 EUR |
| STP | 2008 DHS |
| SVK | 1995 ISSP,1998 ISSP,1999 ISSP,2002 ISSP,2003 ISSP,2003 WHS,2004 EUR,2004 ISSP,2005 EUR,2006 EUR,2007 EUR,2007 ISSP,2008 EUR,2008 ISSP,2009 EUR,2009 ISSP,2010 EUR,2010 ISSP,2011 EUR,2011 ISSP,2012 EUR,2012 ISSP,2013 EUR,2014 EUR,2015 EUR |
| SVN | 1993 ISSP,1994 ISSP,1995 ISSP,1996 ISSP,1997 ISSP,1998 ISSP,1999 ISSP,2000 ISSP,2001 ISSP,2002 ISSP,2003 ISSP,2004 EUR,2004 ISSP,2005 EUR,2005 ISSP,2006 EUR,2006 ISSP,2007 EUR,2007 ISSP,2008 EUR,2008 ISSP,2009 EUR,2009 ISSP,2010 EUR,2010 ISSP,2011 EUR,2011 ISSP,2012 EUR,2012 ISSP,2013 EUR,2014 EUR,2015 EUR |
| SWE | 1994 ISSP,1995 EUR,1995 ISSP,1996 EUR,1996 ISSP,1997 EUR,1997 ISSP,1998 EUR,1998 ISSP,1999 EUR,1999 ISSP,2000 EUR,2000 ISSP,2001 EUR,2002 EUR,2002 ISSP,2003 EUR,2003 ISSP,2003 WHS,2004 EUR,2004 ISSP,2005 EUR,2005 ISSP,2006 EUR,2006 ISSP,2007 EUR,2007 ISSP,2008 EUR,2008 ISSP,2009 EUR,2009 ISSP,2010 EUR,2010 ISSP,2011 EUR,2011 ISSP,2012 EUR,2012 ISSP,2013 EUR,2014 EUR,2015 EUR |
| SWZ | 2003 WHS,2006 DHS |
| TCD | 1996 DHS,2003 WHS,2004 DHS |
| TGO | 1998 DHS,2013 DHS |
| TJK | 2012 DHS |
| TLS | 2009 DHS |
| TUN | 2003 WHS |
| TUR | 1993 DHS,1998 DHS,2003 DHS,2003 WHS,2004 EUR,2005 EUR,2006 EUR,2007 EUR,2008 EUR,2008 ISSP,2009 EUR,2009 ISSP,2010 EUR,2010 ISSP,2011 EUR,2011 ISSP,2012 EUR,2012 ISSP,2013 EUR,2014 EUR,2015 EUR |
| TWN | 2002 ISSP,2003 ISSP,2004 ISSP,2005 ISSP,2006 ISSP,2007 ISSP,2008 ISSP,2009 ISSP,2010 ISSP,2011 ISSP,2012 ISSP |
| TZA | 1996 DHS,1999 DHS,2004 DHS,2009 DHS |
| UGA | 1991 IPUMS,1995 DHS,2000 DHS,2006 DHS,2011 DHS |
| UKR | 2003 WHS,2007 DHS,2008 ISSP,2009 ISSP |
| URY | 1963 IPUMS,1975 IPUMS,1985 IPUMS,1996 IPUMS,2003 ISSP,2003 WHS,2004 ISSP,2006 IPUMS,2006 ISSP,2007 ISSP,2008 ISSP |
| USA | 1960 IPUMS,1970 IPUMS,1980 IPUMS,1989 ISSP,1990 ISSP,1991 ISSP,1992 ISSP,1993 ISSP,1994 ISSP,1995 ISSP,1996 ISSP,1997 ISSP,1998 ISSP,1999 ISSP,2000 ISSP,2001 ISSP,2002 ISSP,2003 ISSP,2004 ISSP,2005 ISSP,2006 ISSP,2007 ISSP,2008 ISSP,2009 ISSP,2010 ISSP,2011 ISSP,2012 ISSP |
| UZB | 1996 DHS |
| VEN | 1971 IPUMS,1981 IPUMS,1990 IPUMS,2001 IPUMS,2006 ISSP,2008 ISSP,2009 ISSP,2012 ISSP |
| VNM | 1989 IPUMS,1997 DHS,1999 IPUMS,2002 DHS,2003 WHS,2009 IPUMS |
| ZAF | 1998 DHS,2003 WHS,2004 ISSP,2005 ISSP,2006 ISSP,2007 ISSP,2008 ISSP,2009 ISSP,2010 ISSP,2011 ISSP,2012 ISSP |
| ZMB | 1992 DHS,1996 DHS,2001 DHS,2003 WHS,2007 DHS,2013 DHS |
| ZWE | 1994 DHS,1999 DHS,2003 WHS,2005 DHS,2010 DHS |

All data sources used in the analysis from which single year education data were attained. Each row represents a single country, specified with 3-character ISO3 codes.

**Table B. Binned Data Sources**

| ISO3 Country Code | Sources Used |
| --- | --- |
| ARG | 2001 IPUMS |
| ARM | 2001 IPUMS,2011 IPUMS |
| AUT | 1971 IPUMS,1981 IPUMS,1991 IPUMS,2001 IPUMS,2011 IPUMS |
| BFA | 1996 IPUMS,2006 IPUMS |
| BGD | 1991 IPUMS,2001 IPUMS,2011 IPUMS |
| BLR | 1999 IPUMS |
| BOL | 2001 IPUMS |
| BRA | 1960 IPUMS,1970 IPUMS,1980 IPUMS,1991 IPUMS,2000 IPUMS,2010 IPUMS |
| CAN | 1971 IPUMS,1981 IPUMS,1991 IPUMS,2001 IPUMS |
| CHE | 1970 IPUMS,1980 IPUMS,1990 IPUMS,2000 IPUMS |
| CHN | 1982 IPUMS,1990 IPUMS |
| COL | 2005 IPUMS |
| CUB | 2002 IPUMS |
| EGY | 2006 IPUMS |
| ESP | 1981 IPUMS,1991 IPUMS,2001 IPUMS,2011 IPUMS |
| ETH | 1984 IPUMS,1994 IPUMS,2007 IPUMS |
| FJI | 1976 IPUMS,1986 IPUMS,1996 IPUMS,2007 IPUMS |
| FRA | 1962 IPUMS,1968 IPUMS,1975 IPUMS,1982 IPUMS,1990 IPUMS,1999 IPUMS,2006 IPUMS,2011 IPUMS |
| GRC | 1971 IPUMS,1981 IPUMS,1991 IPUMS,2001 IPUMS |
| HTI | 1971 IPUMS |
| HUN | 1970 IPUMS,1980 IPUMS,1990 IPUMS,2001 IPUMS |
| IDN | 1971 IPUMS,1976 IPUMS,1980 IPUMS,1985 IPUMS,1990 IPUMS,1995 IPUMS,2000 IPUMS,2005 IPUMS,2010 IPUMS |
| IND | 1987 IPUMS,1993 IPUMS,1999 IPUMS,2004 IPUMS |
| IRL | 1971 IPUMS,1981 IPUMS,1991 IPUMS,1996 IPUMS,2002 IPUMS,2006 IPUMS,2011 IPUMS |
| IRN | 2006 IPUMS |
| IRQ | 1997 IPUMS |
| ISR | 1972 IPUMS,1983 IPUMS,1995 IPUMS |
| ITA | 2001 IPUMS |
| JAM | 1982 IPUMS |
| JOR | 2004 IPUMS |
| KEN | 1969 IPUMS,1979 IPUMS,1989 IPUMS,1999 IPUMS,2009 IPUMS |
| KGZ | 1999 IPUMS,2009 IPUMS |
| KHM | 1998 IPUMS,2008 IPUMS |
| LBR | 2008 IPUMS |
| LCA | 1980 IPUMS,1991 IPUMS |
| MLI | 1987 IPUMS |
| MNG | 1989 IPUMS,2000 IPUMS |
| MOZ | 1997 IPUMS,2007 IPUMS |
| MYS | 1970 IPUMS,1980 IPUMS,1991 IPUMS,2000 IPUMS |
| NGA | 2006 IPUMS,2007 IPUMS,2008 IPUMS,2009 IPUMS,2010 IPUMS |
| PAK | 1973 IPUMS,1981 IPUMS,1998 IPUMS |
| PER | 1993 IPUMS,2007 IPUMS |
| PHL | 1990 IPUMS,1995 IPUMS,2000 IPUMS |
| PRI | 1990 IPUMS,2000 IPUMS,2005 IPUMS |
| PRT | 1981 IPUMS,1991 IPUMS,2001 IPUMS,2011 IPUMS |
| PSE | 1997 IPUMS |
| ROU | 1977 IPUMS,1992 IPUMS,2002 IPUMS |
| SDN | 2008 IPUMS |
| SLE | 2004 IPUMS |
| SSD | 2008 IPUMS |
| SVN | 2002 IPUMS |
| THA | 1970 IPUMS,1980 IPUMS,1990 IPUMS,2000 IPUMS |
| TUR | 1985 IPUMS,1990 IPUMS,2000 IPUMS |
| TZA | 1988 IPUMS,2002 IPUMS |
| UGA | 2002 IPUMS |
| UKR | 2001 IPUMS |
| URY | 2011 IPUMS |
| USA | 1990 IPUMS,2000 IPUMS,2005 IPUMS,2010 IPUMS |
| ZAF | 1996 IPUMS,2001 IPUMS,2007 IPUMS,2011 IPUMS |
| ZMB | 1990 IPUMS,2000 IPUMS,2010 IPUMS |

All data sources used in the analysis from which binned education data were attained. Each row represents a single country, specified with 3-character ISO3 codes.
