# Supplementary material for: Improving the estimation of educational attainment: New methods for assessing average years of schooling from binned data
Source: PLoS One. 2018 Nov 29;13(11):e0208019. doi: 10.1371/journal.pone.0208019 (PMC6264843; doi:10.1371/journal.pone.0208019)
Supplement: S2 File — (DOCX) [file pone.0208019.s002.docx]

**S2 File. Code to Run Predictive Validity Exercise**

In the below section, we provide the code used to run the predictive validity exercise detailed in this study. File paths, file names, and personal network information have been redacted, and replaced with “FILE_PATH”,” FILE”, and “NETWORK_INFO” respectively.

Script 1 – STATA file, prepares holdouts, initiates predictive validity exercise in parallel for each single-year data source

_// Master Script for Bin-Splitting Models Validation Exercise_

_// Creates Holdouts and Launches Validation Process_

_quietly {_

_if c(os) == "Unix" {_

_local prefix "FILE_PATH"_

_set more off_

_set odbcmgr unixodbc_

_}_

_else if c(os) == "Windows" {_

_local prefix "FILE_PATH"_

_}_

_set more off_

_cap restore, not_

_cap set maxvar 20000_

_set seed 80085_

_adopath + "FILE_PATH"_

_local root "FILE_PATH"_

_}_

_local repo `1'_

_local append_all 0_

_local do_holdouts 0_

_// Load all data and append_

_if `append_all' == 1 {_

_import delimited "FILE_PATH/FILE", delim(",") varn(1) clear_

_keep if bin_id == "`bin_id'"_

_tempfile edu_groups_

_save `edu_groups', replace_

_clear_

_local crosswalk_database = "FILE_PATH"_

_local surveys: dir "`crosswalk_database'" files "*", respectcase_

_local max = 0_

_foreach survey of local surveys {_

_local max = `max' + 1_

_}_

_tempfile all_

_save `all', emptyok_

_local count = 0_

_foreach survey of local surveys {_

_di "`count'/`max'"_

_quietly {_

_local count = `count' + 1_

_use "`crosswalk_database'/`survey'", clear_

_gen survey = subinstr("`survey'", ".dta", "", .)_

_append using `all'_

_save `all', replace_

_}_

_}_

_split survey, parse("_")_

_rename survey1 source_

_drop survey*_

_foreach var of varlist * {_

_cap _strip_labels `var'_

_}_

_// Save database of all survey/country/year for selecting holdouts and "closest" training sample_

_preserve_

_quiet run "FILE_PATH/FILE"_

_create_connection_string, server("NETWORK_INFO") database("NETWORK_INFO") user("NETWORK_INFO") password("NETWORK_INFO")_

_local conn_string = r(conn_string)_

_odbc load, exec("NETWORK_INFO") `conn_string' clear_

_tempfile regions_

_save `regions', replace_

_restore_

_preserve_

_keep iso3 year source_

_duplicates drop_

_merge m:1 iso3 using `regions', keep(3) nogen_

_egen survey = concat(iso3 year source), p("_")_

_tempfile all_surveys_

_save `all_surveys', replace_

_save "`FILE_PATH/FILE", replace_

_restore_

_// Save full database for crosswalking_

_save "FILE_PATH/FILE", replace_

_}_

_if `do_holdouts' == 1 {_

_// Make list of available schemas for crosswalk validation_

_local schema_dir = "FILE_PATH"_

_local schemas: dir "`schema_dir'" files "*", respectcase_

_clear_

_tempfile all_schemas_

_save `all_schemas', emptyok_

_foreach schema of local schemas {_

_use "`schema_dir'/`schema'", clear_

_append using `all_schemas'_

_save `all_schemas', replace_

_}_

_gen schema = type + "_" + string(year) + "_" + iso3_

_keep schema_

_duplicates drop_

_gen schema_num = _n_

_local max_schema = _N_

_save `all_schemas', replace_

_// Generate holdout samples (10%)_

_use "FILE_PATH/FILE", clear_

_tempfile full_sample_

_save `full_sample', replace_

_local total_surveys = _N_

_forvalues i = 1/10 {_

_use `full_sample', clear_

_if `i' >= 1 | `i' <= 9 {_

_local this_sample = round(`total_surveys'/10)_

_sample `this_sample', count_

_}_

_gen schema_num = floor((`max_schema'-1+1)*runiform() + 1)_

_merge m:1 schema_num using `all_schemas', keep(3) assert(2 3) nogen_

_save "FILE_PATH/FILE", replace_

_keep survey_

_tempfile sample_

_save `sample', replace_

_use `full_sample', clear_

_merge 1:1 survey using `sample', keep(1) nogen_

_save `full_sample', replace_

_}_

_}_

_// Parallelize crosswalk jobs over specific survey from each holdout sample_

_local run_crosswalk 1_

_local run_regression 1_

_local space_weight = 0_

_forvalues holdout = 1/10 {_

_use "FILE_PATH/FILE, clear_

_tempfile holdout_sample_

_save `holdout_sample', replace_

_levelsof survey, l(surveys)_

_foreach survey of local surveys {_

_use `holdout_sample' if survey == "`survey'", clear_

_local target_schema = schema_

_di "`holdout' `target_schema' `survey'"_

_NETWORK_INFO }}_

Script 2 – R File, runs predictive validity exercise separately for each single-year data source, loops over hyper-parameter sets

##Run Bin-Splitting Model for a Specific Single-Year Dataset Binned with a Specific Schema

## Settings and libraries

root <- ifelse(Sys.info()[1]=="Windows", "FILE_PATH","FILE_PATH")

hroot <- ifelse(Sys.info()[1]=="Windows", "FILE_PATH", "FILE_PATH")

library(data.table)

library(ggplot2)

library(haven)

library(reshape2)

library(plyr)

library(lme4)

library(reldist, lib.loc=paste0(hroot,"/packages"))

setwd(paste0(root,"FILE_PATH"))

root_dir <- paste0(root,"FILE_PATH")

## Logit functions

logit <- function(x) {

log(x/(1-x))

}

invlogit <- function(x) {

exp(x)/(1+exp(x))

}

## Load arguments and set training parameters for validation

print(commandArgs())

target_hold_out = commandArgs()[3]

target_schema = commandArgs()[4]

target_survey = commandArgs()[5]

target_space_weight = commandArgs()[6]

run_crosswalk = commandArgs()[7]

run_regression = commandArgs()[8]

# Set date of run for versioning outputs

run_date <- gsub("-","_",Sys.Date())

# Set sizes to iterate over for crosswalk training sample size

training_sizes <- c(1,2,4,6,8,12,16,20,40,60,80)

## Pull information for this target survey

survey_info <- read_dta(paste0(root_dir,"data/crosswalk/crosswalk_validation/holdout_samples/holdout_",target_hold_out,".dta"))

target_year <- survey_info$year[survey_info$survey==target_survey]

target_iso3 <- survey_info$iso3[survey_info$survey==target_survey]

target_region <- survey_info$region[survey_info$survey==target_survey]

target_super_region <- survey_info$super_region[survey_info$survey==target_survey]

# Save developed status to restrict training data eligible for selection

dev_status <- read_dta(paste0(root_dir,"FILE_PATH/FILE"))

target_dev <- as.numeric(dev_status$developed[dev_status$iso3==target_iso3])

##****************************************************************************************************

##****************************************************************************************************

## Pull target holdout survey from single-year database. Apply schema/proportions to target survey,

## save a copy with true single-year proportion of entire distribution (these sum to 1) and group

## proportions (these sum to 1). Do the same with our entire single-year database minus this

## holdout partition. Calculate spatial and temporal distance in training database.

##****************************************************************************************************

##****************************************************************************************************

print("Set up target data and single-year training database...")

# Load full single-year database

full_database <- as.data.table(read_dta(paste0(root_dir,"FILE_PATH/FILE")))

full_database[, survey := paste0(iso3,"_",year,"_",source)]

names(full_database)[names(full_database) == "edu_yrs"] = "edu_start"

# Pull out target survey

target_data <- full_database[survey==target_survey,]

target_data <- target_data[, c('edu_start','age_start','sex','prop'), with = FALSE]

target_data <- target_data[order(sex,age_start)]

# Pull schema

schema <- as.data.table(read_dta(paste0(root_dir,"FILE_PATH",target_schema,".dta")))

schema <- schema[, c('edu_start','bin','technical','prop','BL'), with = FALSE]

names(schema)[names(schema) == "prop"] = "bin_prop"

# Save total number of bins for output

number_of_bins <- length(unique(schema$bin))

# Merge schema and apply proportions

target_data <- merge(target_data, schema, by='edu_start', all.x = TRUE, allow.cartesian = TRUE)

target_data <- target_data[bin_prop!="NA",]

target_data[, true_single_year_prop := prop * bin_prop]

# Rake to new "total distribution."

new_total <- target_data[, list(new_total=sum(true_single_year_prop)), by=c('age_start','sex')]

target_data <- merge(target_data, new_total, by=c('age_start','sex'))

target_data[, true_single_year_prop := true_single_year_prop / new_total]

# Collapse to single-year

true_single_year_props <- target_data[, list(true_single_year_prop=sum(true_single_year_prop)), by=c('age_start','sex','edu_start')]

true_single_year_props[true_single_year_prop=="NaN", true_single_year_prop := 0]

true_group_props <- target_data[, list(true_group_prop=sum(true_single_year_prop)), by=c('age_start','sex','bin')]

true_group_props[true_group_prop=="NaN", true_group_prop := 0]

# Collapse single-year proportions in target data

bl_estimates <- target_data[, list(bl_single_year_prop=sum(true_single_year_prop)), by=c('age_start','sex','BL')]

bl_estimates[bl_single_year_prop=="NaN", bl_single_year_prop := 0]

names(bl_estimates)[names(bl_estimates) == "BL"] = "edu_start"

bl_estimates[, survey := target_survey]

bl_estimates[, nbins := number_of_bins]

bl_estimates <- bl_estimates[order(sex,age_start,edu_start)]

if(target_space_weight==0) write.csv(bl_estimates, file = paste0(root_dir,"FILE_PATH/",target_hold_out,"_",target_survey,"_",target_schema,".csv"))

# Apply same process to full single-year training database minus holdout partition

training_database <- merge(full_database, dev_status, by="iso3")

training_database <- training_database[developed==target_dev,]

# Drop out holdout data. Drop specific target survey also.

holdout_to_drop <- as.data.table(read_dta(paste0(root_dir,"FILE_PATH/FILE",target_hold_out,".dta")))

holdout_to_drop$drop <- 1

holdout_to_drop <- holdout_to_drop[, c('survey','drop'), with = FALSE]

training_database <- merge(training_database, holdout_to_drop, by='survey', all.x=TRUE)

training_database <- training_database[is.na(training_database$drop),]

training_database$drop <- NULL

training_database <- training_database[survey!=target_survey,]

# Merge schema and apply proportions

training_database <- merge(training_database, schema, by='edu_start', all.x = TRUE, allow.cartesian = TRUE)

training_database <- training_database[bin_prop!="NA",]

training_database[, true_single_year_prop := prop * bin_prop]

# Rake to new "total distribution.

new_total <- training_database[, list(new_total=sum(true_single_year_prop)), by=c('iso3','year','survey','age_start','sex')]

training_database <- merge(training_database, new_total, by=c('iso3','year','survey','age_start','sex'))

training_database[, true_single_year_prop := true_single_year_prop / new_total]

training_database <- training_database[, c('iso3','year','survey','age_start','sex','edu_start','bin','true_single_year_prop'), with = FALSE]

training_database <- training_database[order(iso3,survey,sex,age_start,bin,edu_start)]

# Collapse to bin totals, generate proportion single-year within bin

training_true_group_props <- training_database[, list(true_group_prop=sum(true_single_year_prop)), by=c('iso3','year','survey','age_start','sex','bin')]

training_database <- merge(training_database, training_true_group_props, by=c('iso3','year','survey','age_start','sex','bin'))

training_database[, single_year_wbin_prop := true_single_year_prop / true_group_prop]

training_database[single_year_wbin_prop=="NaN", single_year_wbin_prop := 0]

# Calculate space/time distance for each training survey from this target survey. Normalize space/time distances to be between 0-1.

# Assume equal space "distance" between geographic levels, i.e. the spatial distance between iso3

# and region is equivalent to the spatial distance between region and super region.

# Use range of training dataset to normalize distance over time.

# Merge region, super region names

full_survey_list <- as.data.table(read_dta(paste0(root_dir,"FILE_PATH/FILE")))

full_survey_list <- full_survey_list[, c('survey','region_name','super_region_name'), with = FALSE]

training_database <- merge(training_database, full_survey_list, by='survey', all.x=TRUE)

# Spatial distance

training_database[, spatial_distance := 1]

training_database[iso3==target_iso3, spatial_distance := 0]

training_database[region_name==target_region & spatial_distance==1, spatial_distance := 1/3]

training_database[super_region_name==target_super_region & spatial_distance==1, spatial_distance := 2/3]

# Temporal distance

training_database[, temporal_distance := abs(year - target_year) / (max(year) - min(year))]

##****************************************************************************************************

##****************************************************************************************************

## Define Space-Time Model function.

## Inputs: training size, spatial weight, age, sex, bin, year

## 1. Subset single-year database on age, sex, bin, year.

## 2. Select training sample from single-year subset using training size and weights.

## 3. Crosswalk.

## 4. Apply crosswalk to target data group proportions to get estimated year within bin proportions.

## 5. Save year within bin proportions by age, sex, bin, year, training size, spatial weight.

##****************************************************************************************************

##****************************************************************************************************

if(run_crosswalk==1) {

print("Define crosswalk function and apply...")

crosswalk <- function(x) {

training_size <- gsub(" ", "", x[1])

training_size <- as.numeric(training_size)

space_weight <- as.numeric(target_space_weight)

iage <- as.numeric(x[2])

isex <- as.numeric(x[3])

ibin <- x[4]

iyear <- gsub(" ", "", x[5])

iyear <- as.numeric(iyear)

time_weight <- 1 - space_weight

# Subset training database and calculate distance with weights

training_set <- subset(training_database, age_start == iage)

training_set <- subset(training_set, sex == isex)

training_set <- subset(training_set, bin == ibin)

training_set <- subset(training_set, edu_start == iyear)

training_set[, distance := (spatial_distance * space_weight) + (temporal_distance * time_weight)]

training_set <- training_set[single_year_wbin_prop!="NaN",]

# Pick x closest surveys based on this iteration of training_size

ranks <- training_set[, list(distance=mean(distance,na.rm=TRUE)), by=c("survey")]

ranks <- ranks[order(rank(distance))]

ranks <- ranks[1:training_size,]

ranks$distance <- NULL

training_set <- merge(training_set, ranks, by="survey")

# Transform outcome for model

# Fit model

model <- lm(logit_single_year_wbin_prop ~ 1, training_set)

# Pull parameters and apply to target survey

training_set[, constant := model$coefficients[1]]

training_set <- training_set[, c('edu_start','bin','age_start','sex','constant'), with = FALSE]

training_set <- unique(training_set)

estimates <- merge(training_set, true_group_props, by=c('bin','age_start','sex'))

estimates[, space_weight := space_weight]

estimates[, training_size := training_size]

estimates[, est_single_year_wbin_prop := invlogit(constant)]

estimates[, est_single_year_wbin_prop := constant]

return(estimates)

}

# Make list of parameters using target data for unique bin/year combinations

parameters.df <- target_data[, c('edu_start','bin','age_start','sex'), with = FALSE]

parameters.df[, training_size := 0]

parameters.df.full <- parameters.df

for(training_size in training_sizes) {

i.parameters.df <- parameters.df

i.parameters.df$training_size <- training_size

parameters.df.full <- rbind(parameters.df.full, i.parameters.df)

}

parameters.df.full <- parameters.df.full[training_size!=0,]

setcolorder(parameters.df.full, c('training_size', 'age_start', 'sex', 'bin', 'edu_start'))

parameters.df.full <- as.matrix(parameters.df.full)

parameters.list <- split(parameters.df.full, row(parameters.df.full))

# Apply crosswalk to every combination and append

#op <- pboptions(type="timer")

system.time(all_estimates <- lapply(parameters.list, crosswalk))

#pboptions(op)

all_estimates.df <- do.call(rbind.fill, all_estimates)

##****************************************************************************************************

##****************************************************************************************************

## Append all estimated year within bin proportions.

## Rake estimated year within bin proportions to 1 within bin.

## Merge true group proportions and true single-year proportions from target survey.

## Estimated single-year proportion = (raked year within bin proportion) * (true group proportion)

## Collapse over single-year to account for multiple bins contributing to the same single-year.

## Final output by target survey, training size, spatial weight, age, sex:

## - Estimated single-year proportion

## - True single-year proportion

##****************************************************************************************************

##****************************************************************************************************

print("Format and save...")

# Rake estimated year within bin proportions from crosswalk model to 1 within bin.

all_estimates.dt <- as.data.table(all_estimates.df)

all_estimates.new_totals <- all_estimates.dt[, list(new_total=sum(est_single_year_wbin_prop)), by=c('training_size', 'space_weight', 'age_start', 'sex', 'bin')]

all_estimates.dt <- merge(all_estimates.dt, all_estimates.new_totals, by=c('training_size', 'space_weight', 'age_start', 'sex', 'bin'))

all_estimates.dt[, est_single_year_wbin_prop := est_single_year_wbin_prop / new_total]

all_estimates.dt[est_single_year_wbin_prop=="NaN", est_single_year_wbin_prop := 0]

# Generate estimated single-year proportion

all_estimates.dt[, est_single_year_prop := est_single_year_wbin_prop * true_group_prop]

# collapse over single-year to accounr for multiple bins contributing to same single-year

all_estimates.dt <- all_estimates.dt[, list(est_single_year_prop=sum(est_single_year_prop)), by=c('training_size', 'space_weight', 'age_start', 'sex', 'edu_start')]

# Merge true single-year proportions and save

all_estimates.dt <- merge(all_estimates.dt, true_single_year_props, by=c('age_start', 'sex', 'edu_start'))

all_estimates.dt[, survey := target_survey]

all_estimates.dt[, nbins := number_of_bins]

# Version outputs

dir.create(paste0("FILE_PATH",run_date), showWarnings = FALSE)

space_weight <- gsub("[.]", "", as.character(target_space_weight))

write.csv(all_estimates.dt, file = paste0("FILE_PATH/",run_date,"/",target_hold_out,"_sp",space_weight,"_",target_survey,"_",target_schema,".csv"))

}

##****************************************************************************************************

##****************************************************************************************************

## NESTED MIXED EFFECTS REGRESSION

##****************************************************************************************************

##****************************************************************************************************

if(run_regression==1) {

print("Running LMER regression method...")

# Add necessary variables to true group props of target data

lmer_true_group_props <- true_group_props

lmer_true_group_props$iso3 <- target_iso3

lmer_true_group_props$region_name <- target_region

lmer_true_group_props$year <- target_year

# Transform for logit

training_database <- training_database[single_year_wbin_prop==0, single_year_wbin_prop := 0.0001]

training_database <- training_database[single_year_wbin_prop==1, single_year_wbin_prop := 0.9999]

lmer_crosswalk <- function(x) {

iyear <- gsub(" ", "", x[1])

iyear <- as.numeric(iyear)

ibin <- x[2]

training_set <- subset(training_database, bin == ibin)

training_set <- subset(training_set, edu_start == iyear)

## FIT MODEL

model <- lmer(logit(single_year_wbin_prop) ~ (1|sex/age_start) + (1|region_name/iso3) + year, data=training_set)

estimates <- lmer_true_group_props[bin == ibin,]

estimates[, lmer_single_year_wbin_prop := invlogit(predict(model, data=estimates))]

estimates[, edu_start := iyear]

return(estimates)

}

lmer_parameters <- as.matrix(unique(target_data[, c('edu_start','bin'), with = FALSE]))

lmer_parameters <- split(lmer_parameters, row(lmer_parameters))

system.time(lmer_estimates <- lapply(lmer_parameters, lmer_crosswalk))

lmer_estimates <- do.call(rbind.fill, lmer_estimates)

# Rake estimated year within bin proportions from crosswalk model to 1 within bin.

lmer_estimates.dt <- as.data.table(lmer_estimates)

lmer_estimates.new_totals <- lmer_estimates.dt[, list(new_total=sum(lmer_single_year_wbin_prop)), by=c('age_start', 'sex', 'bin')]

lmer_estimates.dt <- merge(lmer_estimates.dt, lmer_estimates.new_totals, by=c('age_start', 'sex', 'bin'))

lmer_estimates.dt[, lmer_single_year_wbin_prop := lmer_single_year_wbin_prop / new_total]

lmer_estimates.dt[lmer_single_year_wbin_prop=="NaN", lmer_single_year_wbin_prop := 0]

# Generate estimated single-year proportion

lmer_estimates.dt[, lmer_single_year_prop := lmer_single_year_wbin_prop * true_group_prop]

# Collapse over single-year to account for multiple bins contributing to same single-year

lmer_estimates.dt <- lmer_estimates.dt[, list(lmer_single_year_prop=sum(lmer_single_year_prop)), by=c('age_start', 'sex', 'edu_start')]

# Merge true single-year proportions and save.

lmer_estimates.dt <- merge(lmer_estimates.dt, true_single_year_props, by=c('age_start', 'sex', 'edu_start'))

lmer_estimates.dt[, survey := target_survey]

lmer_estimates.dt[, nbins := number_of_bins]

# Version outputs

dir.create(paste0("FILE_PATH",run_date), showWarnings = FALSE)

dir.create(paste0("FILE_PATH",run_date,"FILE_PATH"), showWarnings = FALSE)

write.csv(lmer_estimates.dt, file = paste0("FILE_PATH",run_date,"/FILE_PATH/",target_hold_out,"_",target_survey,"_",target_schema,".csv"))

}

## END ***********************************************************************************************

Script 3 – R File, combines results from script 2, calculates predictive validity statistics

library(parallel)

library(data.table)

library(ggplot2)

library(haven)

library(SDMTools)

jpath <- ifelse(Sys.info()[1]=="Windows", "FILE_PATH", "FILE_PATH")

cores <- 40

model_version <- "FILE_PATH"

##Append Standard Duration results

input.path <- paste0(model_version,"BL/")

file.list <- list.files(input.path,pattern="csv")

ptm <- proc.time()

bldata.list <- mclapply(file.list, function(file) {

bldata <- fread(paste0(input.path, file))

},mc.cores=ifelse(Sys.info()[1]=="Windows", 1, cores))

bldata <- rbindlist(bldata.list)

print(proc.time() - ptm)

print("done appending")

save(bldata,file=paste0(model_version,"FILE_PATH/FILE"))

##Append Mixed Effect model crosswalk results

input.path <- paste0(model_version,"FILE_PATH")

file.list <- list.files(input.path,pattern="csv")

ptm <- proc.time()

medata.list <- mclapply(file.list, function(file) {

medata <- fread(paste0(input.path, file))

#print(file)

},mc.cores=ifelse(Sys.info()[1]=="Windows", 1, cores))

medata <- rbindlist(medata.list)

print(proc.time() - ptm)

print("done appending")

save(medata,file=paste0(model_version,"FILE_PATH/FILE"))

##Append Space-Time Distance results

input.path <- paste0(model_version,"FILE_PATH")

file.list <- list.files(input.path,pattern="csv")

ptm <- proc.time()

data.list <- mclapply(file.list, function(file) {

data <- fread(paste0(input.path, file))

},mc.cores=ifelse(Sys.info()[1]=="Windows", 1, cores))

data <- rbindlist(data.list)

print(proc.time() - ptm)

print("done appending")

save(data,file=paste0(model_version,"FILE_PATH/FILE"))

#Bin Pooling function

binpool <- function(dat) {

dat[dat%in% c(5)] <- 4

dat[dat %in% c(8,9,10,11,12,13)] <- 8

dat[dat %in% c(15,17,18)] <- 15

dat = as.character(dat)

dat[dat == "4"] <- "4-5"

dat[dat == "8"] <- "8-13"

dat[dat == "15"] <- "15-18"

dat = factor(dat,levels=c("4-5","6","7","8-13","14","15-18"))

return(dat)}

#Load Data

load(file="FILE_PATH/FILE")

load(file="FILE_PATH/FILE")

load(file="FILE_PATH/FILE")

#Pool Bins in Data

data$nbins <- binpool(data$nbins)

bldata$nbins <- binpool(bldata$nbins)

medata$nbins <- binpool(medata$nbins)

#Make Mean and SDs of Education

aggs <- data[,list(mean_true=sum(true_single_year_prop * edu_start),mean_est=sum(est_single_year_prop * edu_start),

sd_true = wt.sd(edu_start,true_single_year_prop),sd_est= wt.sd(edu_start,est_single_year_prop)),

by=list(age_start,sex,training_size,space_weight,survey,nbins )]

head(aggs)

aggs_bl <- bldata[,list(mean_est=sum(bl_single_year_prop * edu_start,na.rm=T),

sd_est=wt.sd(edu_start,bl_single_year_prop))

,by=list(age_start,sex,survey,nbins )]

aggs_me <- medata[,list(mean_est=sum(lmer_single_year_prop * edu_start,na.rm=T),

sd_est=wt.sd(edu_start,lmer_single_year_prop)),

by=list(age_start,sex,survey,nbins )]

#Merge on true aggs to standard duration and me estimates

true_aggs <- aggs[aggs$training_size==max(aggs$training_size) & aggs$space_weight==max(aggs$space_weight),]

true_aggs <- true_aggs[, which(grepl("est|training|space", colnames(true_aggs))):=NULL]

aggs_bl <- merge(aggs_bl,true_aggs,by=c("age_start","survey","sex","nbins"))

aggs_me <- merge(aggs_me,true_aggs,by=c("age_start","survey","sex","nbins"))

#Append All Aggregates

aggs$Model <- 'Space-Time Distance'

aggs_bl$Model <- 'Standard Duration'

aggs_me$Model <- 'ME Crosswalk'

c_aggs <- rbind(aggs,aggs_bl,aggs_me,fill=T)

#Calculate RMSE and Median Error

pv <- c_aggs[,list(rmse = sqrt((mean((mean_true - mean_est)^2,na.rm=T))),

mederr = median(mean_est - mean_true),

rmse_sd = sqrt((mean((sd_true - sd_est)^2,na.rm=T))),

mederr_sd = median(sd_est - sd_true,na.rm=T))

,by=list(training_size,space_weight,Model)]

#Over number of bins

pv_bins <- c_aggs[,list(rmse = sqrt((mean((mean_true - mean_est)^2,na.rm=T))),

mederr = median(mean_est - mean_true),

rmse_sd = sqrt((mean((sd_true - sd_est)^2,na.rm=T))),

mederr_sd = median(sd_est - sd_true,na.rm=T)),

by=list(training_size,space_weight,Model,nbins)]

#Over True Mean Attainment

c_aggs[,mean_inc:= floor(mean_true)]

c_aggs[,obs:=1]

pv_meanincs <- c_aggs[,list(surv.obs=length(unique(survey)), rmse = sqrt((mean((mean_true - mean_est)^2,na.rm=T))),

mederr = median(mean_est - mean_true),

rmse_sd = sqrt((mean((sd_true - sd_est)^2,na.rm=T))),

mederr_sd = median(sd_est - sd_true,na.rm=T))

,by=list(training_size,space_weight,Model,mean_inc)]
