# Supplementary material for: Improving the estimation of educational attainment: New methods for assessing average years of schooling from binned data
Source: PLoS One. 2018 Nov 29;13(11):e0208019. doi: 10.1371/journal.pone.0208019 (PMC6264843; doi:10.1371/journal.pone.0208019)
Supplement: S1 Table — Exact values of data shown in Fig 1. Predictive validity metrics for the mean and standard deviation of attainment by number of bins present in the binning schema used. Space-time distance model results shown using hyper-parameter set with optimal RMSE in mean attainment. (DOCX) [file pone.0208019.s003.docx]

**S1 Table. Predictive Validity by Number of Bins**

| Model | Number of Bins | RMSE in Mean | Median Error in Mean | RMSE in SD | Median Error in SD |
| --- | --- | --- | --- | --- | --- |
| Space-Time Distance | 4-5 | 0.29 | 0.00 | 0.34 | -0.04 |
| Standard Duration | 4-5 | 1.22 | -1.10 | 0.66 | 0.27 |
| Nested Mixed Effects | 4-5 | 0.53 | -0.19 | 0.65 | 0.26 |
| Space-Time Distance | 6 | 0.32 | 0.00 | 0.36 | -0.03 |
| Standard Duration | 6 | 1.24 | -1.00 | 0.74 | 0.29 |
| Nested Mixed Effects | 6 | 0.64 | -0.17 | 0.68 | 0.24 |
| Space-Time Distance | 7 | 0.22 | 0.00 | 0.29 | -0.03 |
| Standard Duration | 7 | 1.02 | -0.85 | 0.58 | 0.23 |
| Nested Mixed Effects | 7 | 0.40 | -0.13 | 0.44 | 0.10 |
| Space-Time Distance | 8-13 | 0.36 | 0.00 | 0.31 | -0.01 |
| Standard Duration | 8-13 | 0.90 | -0.13 | 0.86 | 0.33 |
| Nested Mixed Effects | 8-13 | 0.57 | 0.00 | 0.47 | 0.11 |
| Space-Time Distance | 14 | 0.25 | 0.00 | 0.26 | 0.00 |
| Standard Duration | 14 | 0.41 | 0.02 | 0.55 | 0.12 |
| Nested Mixed Effects | 14 | 0.39 | 0.02 | 0.39 | 0.13 |
| Space-Time Distance | 15-18 | 0.12 | 0.00 | 0.14 | 0.00 |
| Standard Duration | 15-18 | 0.17 | -0.01 | 0.19 | 0.00 |
| Nested Mixed Effects | 15-18 | 0.20 | 0.00 | 0.25 | 0.00 |

Exact values of data shown in fig 1**.** Predictive validity metrics for the mean and standard deviation of attainment by number of bins present in the binning schema used. Space-time distance model results shown using hyper-parameter set with optimal RMSE in mean attainment.
