# Supplementary material for: Improving the estimation of educational attainment: New methods for assessing average years of schooling from binned data
Source: PLoS One. 2018 Nov 29;13(11):e0208019. doi: 10.1371/journal.pone.0208019 (PMC6264843; doi:10.1371/journal.pone.0208019)
Supplement: S2 Table — Exact values of data shown in Fig 2. Predictive validity metrics for the mean and standard deviation of attainment by one-year increments of true mean attainment in the population. Space-time distance model results shown using hyper-parameter set with optimal RMSE in mean attainment. (DOCX) [file pone.0208019.s004.docx]

**S2 Table. Predictive Validity by True Mean.**

| Model | True Mean | RMSE in Mean | Median Error in Mean | RMSE in SD | Median Error in SD |
| --- | --- | --- | --- | --- | --- |
| Space-Time Distance | 0 | 0.28 | 0.00 | 0.74 | 0.00 |
| Standard Duration | 0 | 0.97 | 0.00 | 0.66 | -0.09 |
| Nested Mixed Effects | 0 | 0.53 | 0.00 | 0.71 | 0.00 |
| Space-Time Distance | 1 | 0.32 | 0.00 | 0.38 | 0.00 |
| Standard Duration | 1 | 0.70 | 0.00 | 0.53 | -0.09 |
| Nested Mixed Effects | 1 | 0.38 | 0.00 | 0.59 | 0.03 |
| Space-Time Distance | 2 | 0.30 | 0.00 | 0.30 | 0.00 |
| Standard Duration | 2 | 0.49 | 0.00 | 0.45 | -0.05 |
| Nested Mixed Effects | 2 | 0.45 | 0.00 | 0.63 | 0.03 |
| Space-Time Distance | 3 | 0.30 | 0.00 | 0.26 | 0.00 |
| Standard Duration | 3 | 0.38 | 0.00 | 0.50 | -0.02 |
| Nested Mixed Effects | 3 | 0.56 | 0.00 | 0.72 | 0.02 |
| Space-Time Distance | 4 | 0.29 | 0.00 | 0.26 | 0.00 |
| Standard Duration | 4 | 0.42 | -0.03 | 0.43 | 0.00 |
| Nested Mixed Effects | 4 | 0.60 | 0.00 | 0.69 | 0.02 |
| Space-Time Distance | 5 | 0.30 | 0.00 | 0.29 | 0.00 |
| Standard Duration | 5 | 0.55 | -0.10 | 0.41 | 0.00 |
| Nested Mixed Effects | 5 | 0.67 | 0.00 | 0.66 | 0.00 |
| Space-Time Distance | 6 | 0.29 | 0.00 | 0.30 | 0.00 |
| Standard Duration | 6 | 0.69 | -0.27 | 0.43 | 0.05 |
| Nested Mixed Effects | 6 | 0.67 | 0.00 | 0.67 | 0.01 |
| Space-Time Distance | 7 | 0.27 | 0.00 | 0.30 | -0.01 |
| Standard Duration | 7 | 0.81 | -0.46 | 0.55 | 0.13 |
| Nested Mixed Effects | 7 | 0.59 | 0.00 | 0.62 | 0.01 |
| Space-Time Distance | 8 | 0.25 | 0.00 | 0.31 | -0.01 |
| Standard Duration | 8 | 0.94 | -0.59 | 0.62 | 0.21 |
| Nested Mixed Effects | 8 | 0.49 | 0.00 | 0.55 | 0.01 |
| Space-Time Distance | 9 | 0.24 | 0.00 | 0.27 | -0.01 |
| Standard Duration | 9 | 1.00 | -0.69 | 0.64 | 0.29 |
| Nested Mixed Effects | 9 | 0.43 | 0.00 | 0.48 | 0.01 |
| Space-Time Distance | 10 | 0.23 | 0.00 | 0.27 | 0.00 |
| Standard Duration | 10 | 0.97 | -0.68 | 0.66 | 0.30 |
| Nested Mixed Effects | 10 | 0.40 | -0.02 | 0.44 | 0.05 |
| Space-Time Distance | 11 | 0.21 | 0.00 | 0.24 | 0.00 |
| Standard Duration | 11 | 0.95 | -0.65 | 0.67 | 0.32 |
| Nested Mixed Effects | 11 | 0.38 | -0.03 | 0.39 | 0.07 |
| Space-Time Distance | 12 | 0.21 | 0.00 | 0.23 | 0.00 |
| Standard Duration | 12 | 0.92 | -0.60 | 0.63 | 0.25 |
| Nested Mixed Effects | 12 | 0.40 | -0.06 | 0.41 | 0.14 |
| Space-Time Distance | 13 | 0.24 | -0.01 | 0.22 | -0.02 |
| Standard Duration | 13 | 0.93 | -0.62 | 0.58 | 0.16 |
| Nested Mixed Effects | 13 | 0.46 | -0.06 | 0.43 | 0.27 |
| Space-Time Distance | 14 | 0.27 | -0.04 | 0.23 | -0.04 |
| Standard Duration | 14 | 1.00 | -0.81 | 0.64 | 0.14 |
| Nested Mixed Effects | 14 | 0.46 | -0.11 | 0.44 | 0.30 |
| Space-Time Distance | 15 | 0.33 | -0.11 | 0.27 | -0.08 |
| Standard Duration | 15 | 1.16 | -1.11 | 0.69 | 0.16 |
| Nested Mixed Effects | 15 | 0.47 | -0.18 | 0.46 | 0.28 |
| Space-Time Distance | 16 | 0.51 | -0.24 | 0.37 | -0.19 |
| Standard Duration | 16 | 1.41 | -1.45 | 0.72 | 0.17 |
| Nested Mixed Effects | 16 | 0.62 | -0.22 | 0.49 | 0.16 |
| Space-Time Distance | 17 | 0.84 | -0.33 | 0.75 | -0.38 |
| Standard Duration | 17 | 1.68 | -1.72 | 0.63 | 0.17 |
| Nested Mixed Effects | 17 | 0.84 | -0.23 | 0.79 | -0.14 |

Exact values of data shown in fig 2. Predictive validity metrics for the mean and standard deviation of attainment by one-year increments of true mean attainment in the population. Space-time distance model results shown using hyper-parameter set with optimal RMSE in mean attainment.
