# Supplementary material for: Improving the estimation of educational attainment: New methods for assessing average years of schooling from binned data
Source: PLoS One. 2018 Nov 29;13(11):e0208019. doi: 10.1371/journal.pone.0208019 (PMC6264843; doi:10.1371/journal.pone.0208019)
Supplement: S3 Table — (DOCX) [file pone.0208019.s005.docx]

**S3 Table. Predictive Validity of Weighted Regression Space-Time Distance Approach.**

| **Model** | RMSE in Mean | RMSE in SD | Median Error in Mean | Median Error in SD |
| --- | --- | --- | --- | --- |
| Space-Time Distance (Unweighted) | 0.2601 | 0.2861 | <0.0001 | -0.0090 |
| **Space-Time Distance (Weighted)** | **0.3546** | **0.3243** | **.0236** | **-.01572** |
| Nested Mixed Effects | 0.4693 | 0.4987 | -0.0207 | 0.1034 |
| Standard Duration | 0.9235 | 0.6113 | -0.4683 | 0.1403 |

The results of a sensitivity analysis testing the effect of using a space-time distance crosswalk approach that weights training data by distance, as opposed to weighting all training data points equally. This entailed modifying the regression model shown in equation 4 of the main text to use employ weights equal to the distance between each training data point and the binned data being split into bins. Values shown above for the hyper-parameter set showing the best performance in RMSE of mean attainment. This model did not result in an improvement in predictive validity, although it still performed better than the nested mixed effects model or the standard duration approach.
